# Rising coastal wave hazards from climate-driven winds and tropical cyclones

**DOI:** 10.1093/nsr/nwag528

**Published:** 2026-08-22

**Authors:** Ruizi Shi, Jinzhong Min, Changming Dong, Rong-Hua Zhang, Fanghua Xu

**Affiliations:** State Key Laboratory of Climate System Prediction and Risk Management/Key Laboratory of Meteorological Disaster, Ministry of Education/Collaborative Innovation Center on Forecast and Evaluation of Meteorological Disasters/Earth System Modeling Center/School of Atmospheric Sciences/School of Marine Sciences, Nanjing University of Information Science and Technology, Nanjing 210044, China; State Key Laboratory of Climate System Prediction and Risk Management/Key Laboratory of Meteorological Disaster, Ministry of Education/Collaborative Innovation Center on Forecast and Evaluation of Meteorological Disasters/Earth System Modeling Center/School of Atmospheric Sciences/School of Marine Sciences, Nanjing University of Information Science and Technology, Nanjing 210044, China; State Key Laboratory of Climate System Prediction and Risk Management/Key Laboratory of Meteorological Disaster, Ministry of Education/Collaborative Innovation Center on Forecast and Evaluation of Meteorological Disasters/Earth System Modeling Center/School of Atmospheric Sciences/School of Marine Sciences, Nanjing University of Information Science and Technology, Nanjing 210044, China; State Key Laboratory of Climate System Prediction and Risk Management/Key Laboratory of Meteorological Disaster, Ministry of Education/Collaborative Innovation Center on Forecast and Evaluation of Meteorological Disasters/Earth System Modeling Center/School of Atmospheric Sciences/School of Marine Sciences, Nanjing University of Information Science and Technology, Nanjing 210044, China; Department of Earth System Science, Ministry of Education Key Laboratory for Earth System Modeling, Institute for Global Change Studies, Tsinghua University, Beijing 100084, China

**Keywords:** wave storm, enhanced wave hazard, wind-wave interaction, tropical cyclone

## Abstract

Coastal wave storms are intense, long-lasting hazards that threaten populated shorelines worldwide. Under global warming, large-scale wind patterns are changing and modifying ocean surface waves. However, the long-term evolution of coastal wave storms and the drivers of their change remain poorly quantified on global scales. Here, using global wave simulations with corrected tropical-cyclone winds for 1990–2023, we show that coastal wave storm frequency increases worldwide, with the strongest rise along the Atlantic coasts (∼5% per decade). This basin-wide enhancement is primarily driven by intensified nearshore winds, while tropical cyclones act as an additional amplifier. Combining changes in frequency, duration and intensity, we identify the Yellow Sea-East China Sea and the Northwest Atlantic coasts as emerging hotspots of rapidly intensifying wave hazards. Our findings provide the first global-scale baseline for attributing coastal wave-storm trends to changes in the wind-storm environment, thereby informing early-warning systems and guiding adaptation strategies under continued climate change.

## Teaser

Extreme coastal wave hazards have become more frequent worldwide, driven primarily by changes in background winds and further amplified at the basin scale by tropical cyclones.

## INTRODUCTION

Under global warming, changes in large-scale atmospheric and oceanic circulation are reshaping the global wind environment and storm statistics [1–7], with important consequences for the upper ocean and coastal risk [8–11]. Among these, wave storms stand out as a critical yet underappreciated component of compound coastal hazards (Fig. 1), here being defined as long-lasting (≥12 hours) events during which significant wave heights (SWHs) exceed the local 95th percentile of historical record [12–18]. These high-energy events interact synergistically with astronomical tides to amplify storm surge impacts [19], while independently accounting for more than 50% of extreme coastal water levels in many vulnerable regions [20–26]. Despite their significance, wave storms remain poorly constrained in coastal hazard assessments due to the limited ability of traditional tide-gauge observations in isolating and quantifying wave effects [18,27–30]. This critical knowledge gap hinders robust characterization of present and future coastal flood risk [31], particularly in an era of accelerating sea-level rise and evolving storm statistics under global warming effects [5,32–34].

**Figure 1.**
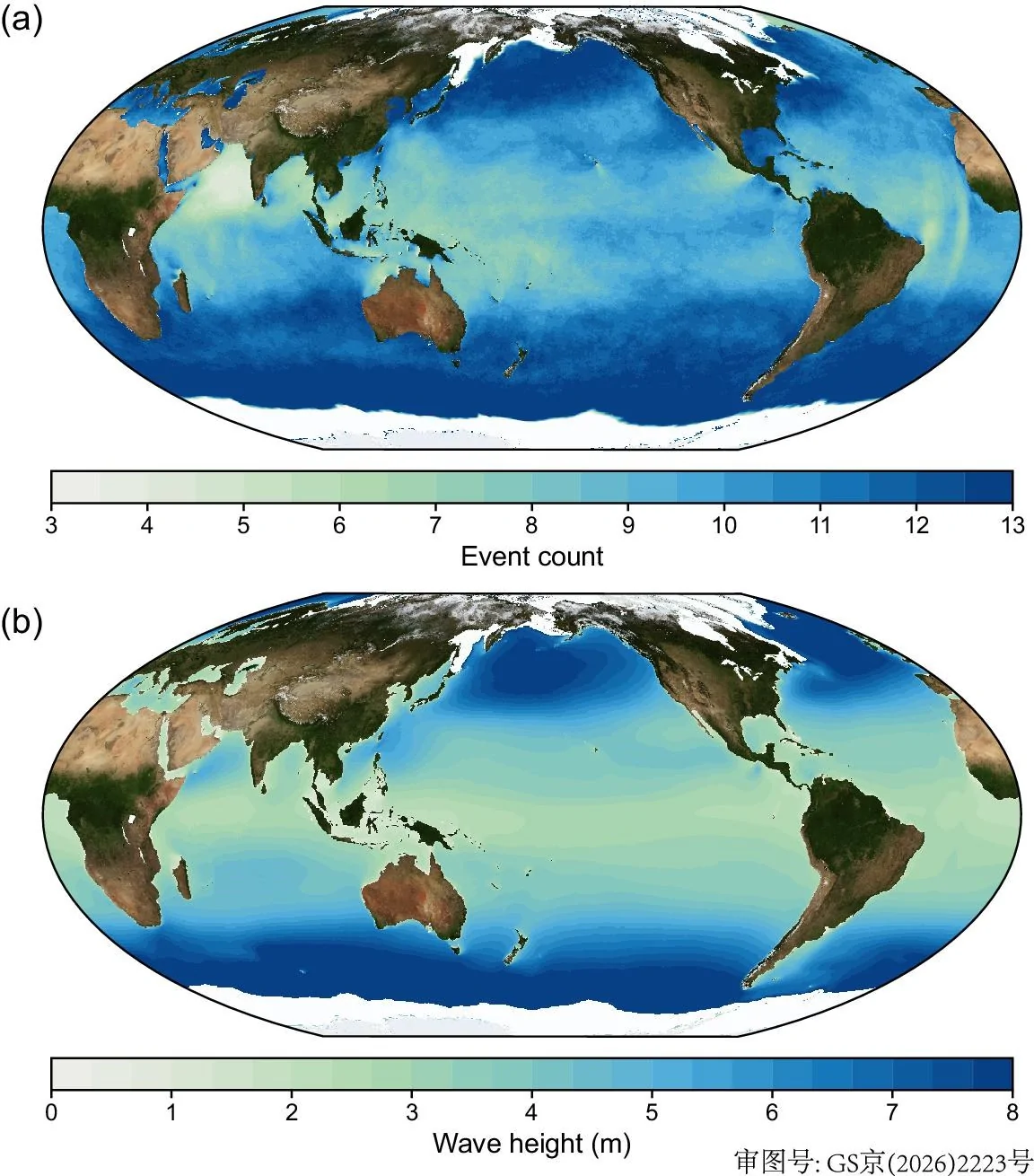
Global distribution of annual wave storm events. Annual mean of (a) event count and (b) event-averaged significant wave height (SWH) calculated from SWH simulated by experiment ‘TCWIND’ using WAVEWATCH III.

Regional studies have shown that even subtle changes in wave storm event (WSE) characteristics (including intensity, frequency and duration) can substantially amplify coastal erosion and flooding [35–37], underscoring the urgent need for detailed assessments of coastal WSE climate variability. Despite its importance, comprehensive global assessments of long-term WSE variability remain scarce. At the same time, the extreme wave height, a key contributor to WSEs, has been extensively examined at the global scale, but reported trends differ markedly in both magnitude and sign, largely because of inconsistencies among datasets [38–43]. These discrepancies mainly reflect data limitations: satellite remote sensing, though relatively reliable, provides temporally and spatially sparse records [44], whereas global reanalysis products can still misrepresent storm-related wind extremes, particularly those associated with tropical cyclones (TCs) [45], with intensity biases reaching up to ∼30% [46–49], thereby severely constraining simulations of storm-driven extreme waves [27].

Recent studies using global wave simulations forced by TC-corrected reanalysis winds reveal that TCs enhance extreme wave heights in the tropics by approximately 35–50% [27]. Consistent analyses further indicate global increases of about 3% per decade in maximum TC wave height and 6% per decade in the spatial extent of TC wave footprints [8]. This body of evidence strongly suggests that TCs must be considered as part of the broader wind forcing that shapes WSEs, yet their quantitative contribution effects to the frequency, duration and intensity of these hazard events, as well as their long-term changes, remain poorly constrained. Given the still-uncertain evolution of TC-related coastal wave hazards, comprehensive global assessments of long-term WSE variability are therefore critical for advancing understanding of how climate change influences coastal hazards.

In this study, we quantify how the evolving wind forcing, including TC-related wind extremes, shapes coastal WSEs (see Methods for definition), including their frequency, duration, intensity and integrated storm wave energy, and assess their long-term evolution over 1990–2023. We perform twin 0.25° global ocean wave simulations under two forcing configurations, with TC (‘TCWIND’) and without TC effects (‘FILT’). The wave model is forced by bias-adjusted 10-m wind fields from the Japanese 55-year Reanalysis (1990–2023) [50], combined with a parametric TC vortex model based on TC best-track observations [51] to provide more realistic wind fields both near and far from TC centers (see Methods for details). Our findings reveal that coastal wave hazards have increased in frequency across most ocean basins, with a global rise of 1.9 ± 0.8% per decade relative to the 34-year mean. This basin-scale growing threat is primarily associated with strengthened nearshore winds, while TCs further reshape its seasonal structure and regional distribution by reducing the winter dominance of WSE occurrence and increasing the summer and autumn shares in TC-prone regions. Trends in event duration and intensity further exhibit strong basin-specific variability, reflecting differing contributions from background winds and TCs.

## RESULTS

### Nearshore wind stress shapes coastal wave storm trends

To evaluate the performance of the simulations, results from WAVEWATCH III (see Methods) are compared with satellite altimeter and buoy observations (Fig. 2 and Fig. S1). The ‘TCWIND’ experiment reproduces observed SWHs with high fidelity, particularly for extreme waves, with a higher correlation (0.99) and a lower root-mean-square error (0.25 m) for the 95th percentile SWH (Fig. S1b). Crucially, the ‘TCWIND’ experiment accurately captures wave states under TC influence (bias: 0.30 m; Fig. 2), while both the ‘FILT’ and ‘JRA’ experiments (see Methods) underestimate them (bias: −1.04 and −0.50 m), highlighting the importance of explicit TC forcing for reliable assessments of wave-related coastal hazards.

**Figure 2.**
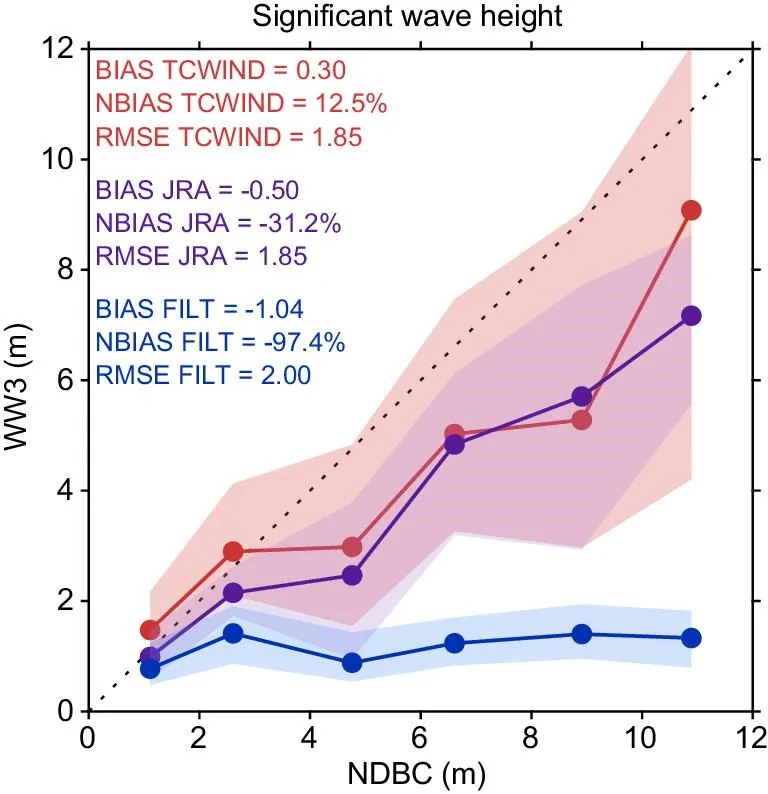
Validation of significant wave heights (SWHs) affected by tropical cyclones (TCs). WW3 simulations of ‘TCWIND’ (red), ‘JRA’ (purple) and ‘FILT’ (blue) experiments versus the National Data Buoy Center coastal observations of SWH at locations affected by TCs (within 4° of the TC centers that experience TC snapshots within 24 hours). The dots correspond to the median values for each bin, while the shaded area represents the 25^th^–75^th^ percentile range. A total of 71 207 valid National Data Buoy Center records are used.

Globally, although TCs exert pronounced impacts in TC-prone tropical and subtropical coastal regions (shaded in Fig. 3a), accounting for over 20% of annual coastal WSE occurrences there, the broader seasonal structure of coastal WSEs is largely set by background wind forcing (Fig. S2). In the ‘FILT’ experiment, WSEs that are not associated with TCs primarily occur during local winters (Fig. S2e-h), and are likely driven by extratropical storms and cold surges linked to the winter monsoon. The exception is the North Indian Ocean, where non-TC-related WSEs primarily occur in summer because the region is strongly governed by the South Asian monsoon, with the strongest winds occurring during boreal summer [52]. In TC-prone tropical and subtropical coasts, this background seasonality is further modified by TC-related wind extremes (Fig. 3b-i), as quantified in the following section.

**Figure 3.**
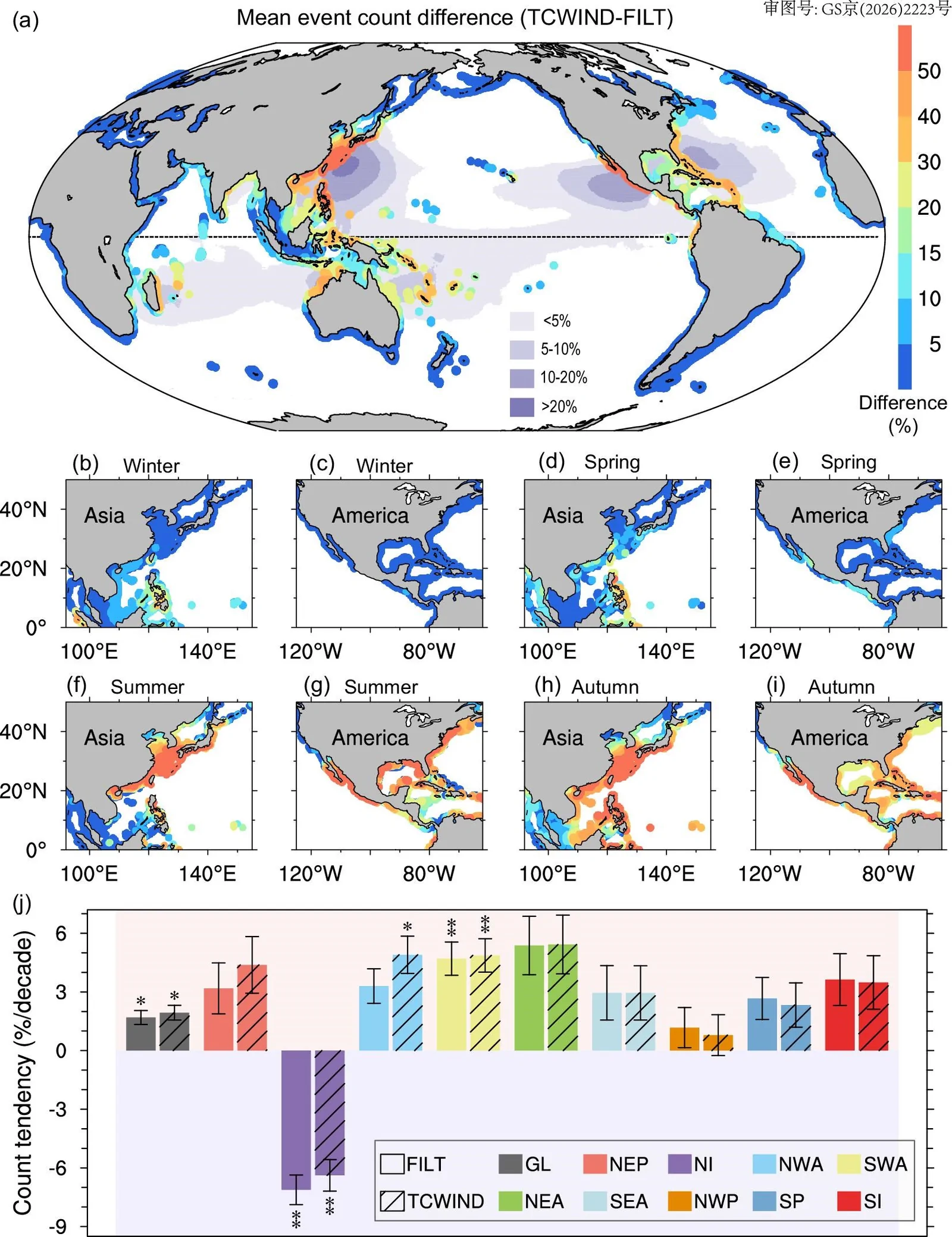
Global contribution of Tropical Cyclones to coastal wave storm events. (a) Annual mean relative difference (%) in event count between the experiment ‘TCWIND’ and ‘FILT’ over 1990–2023, expressed relative to ‘TCWIND’; (b–i) corresponding seasonal count differences for winter, spring, summer and autumn; (j) the area-averaged trend (per decade) of wave storm count in different basins. Purple shading in (a) shows the corresponding relative difference in annual-mean 95^th^-percentile SWH, expressed relative to ‘TCWIND’. GL, NEP, NI, NWA, SWA, NEA, SEA, NWP, SP and SI in (j) represent the globe, the Northeast Pacific, the North Indian Ocean, the Northwest Atlantic, the Southwest Atlantic, the Northeast Atlantic, the Southeast Atlantic, the Northwest Pacific, the South Pacific and the South Indian Ocean, respectively. Basin definitions are provided in Table S1. One and two asterisks in (j) indicate statistical significance of the trend at the 95% and 99% confidence levels based on Student’s t-test, and error bars indicate the standard error of the trend. For the ten regions in (j), the differences between the ‘TCWIND’ and ‘FILT’ trends are statistically significant at the 90% confidence level only for NI and NWA, based on linear regression of the annual ‘TCWIND’ minus ‘FILT’ difference series.

Analysis of linear trends from 1990–2023 reveals a significant global increase in coastal WSE occurrence frequency of 1.9 ± 0.8% per decade (*P* < 0.05) in the ‘TCWIND’ experiment relative to the 34-year mean (Table S1). Although modest in magnitude, this increase is meaningful because WSEs are rare high-threshold events and many coastal regions experience only a few events per year (Fig. 1). The reliability of the simulated wave-field trends is supported by satellite altimetry, with a significant spatial correlation of 0.70 between simulated and observed SWH trends (Fig. S3).

The increase in coastal WSE occurrence frequency is evident across most ocean basins in both experiments (Fig. 3j; Table S1), with the strongest rise along the Atlantic coasts (about 5% per decade). This enhancement is primarily driven by strengthened nearshore wind stress (Fig. S4a and b), likely resulting from amplified land-ocean thermal contrast and poleward shift and intensification of the extratropical cyclones under global warming [7,53,54]. Such poleward shift allows cyclones to travel farther along their northeastward downstream pathways, thereby elongating storm activity toward the northeastern ocean boundaries, and increasing extreme nearshore winds [53,54]. Meanwhile, the North Indian Ocean remains an exception, showing an opposite decreasing trend in coastal WSE occurrence frequency (Fig. 3j). This decline is linked to the weakening of summer monsoon winds over the North Indian Ocean (Fig. S4e and f), caused by rapid warming of the Indian Ocean and relatively subdued warming over the South Asian subcontinent, which together reduce the land-sea thermal gradient [55,56].

Because the strengthening of global coastal wind stress is more likely associated with an increasing occurrence of sudden gusts rather than sustained strong winds, no significant trends are detected for WSE duration or mean SWH globally. This is indicated by the significant widening of the difference between daily maximum and daily mean wind stress (Fig. S4c and d), which occurs mainly over regions where nearshore wind stress also strengthens (Fig. S4a and b). The similar patterns in the ‘TCWIND’ and ‘FILT’ experiments are expected because this diagnostic is dominated by the shared background wind variability rather than by the TC-related wind component. Regional departures from this global pattern are substantial (Fig. 4c-f; Table S1), while they are mainly linked to TC-related effects and are therefore discussed in the next section.

**Figure 4.**
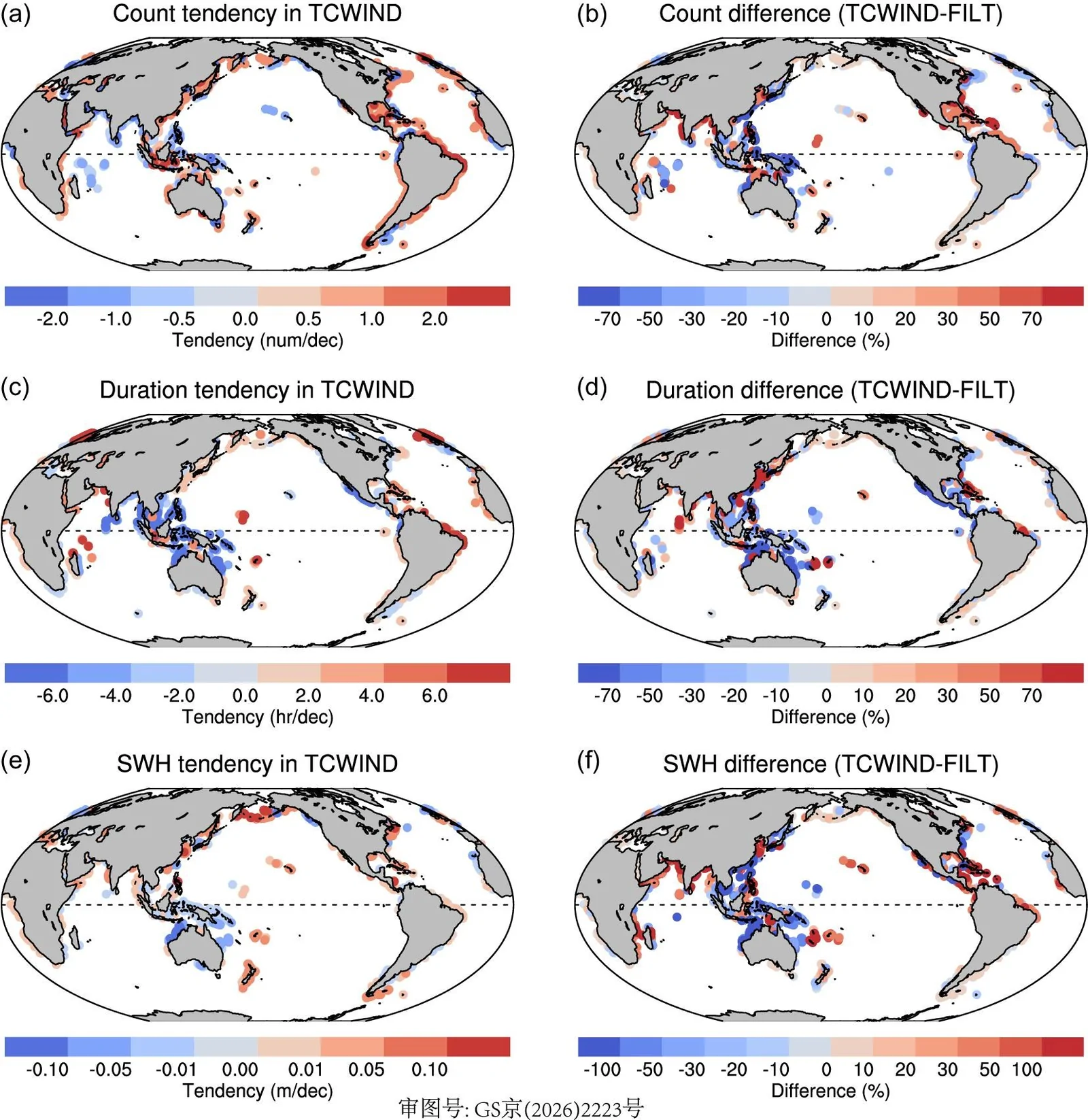
Changes in coastal wave storm events. Linear trends (per decade) in the (a) count, (c) duration and (e) event-averaged SWH of wave storms in global coastal areas for the experiment ‘TCWIND’, over 1990–2023; (b, d and f) the corresponding relative trend differences (%) between the experiment ‘TCWIND’ and ‘FILT’. Percentages are defined as the differences between ‘TCWIND’ and ‘FILT’, expressed relative to the absolute values of the ‘TCWIND’ trends. Only statistically significant trends above the 90% confidence level are shown, based on Student’s *t*-test.

### Tropical cyclones amplify and reshape coastal wave storm hazards

TC influence peaks along the extratropical coasts of the North Pacific, where TCs are most frequent and contribute to more than 50% of WSEs (Fig. 3a). In these regions, the ‘TCWIND’ experiment also shows mean event durations more than 40% higher than those in ‘FILT’ experiment (Fig. S5b), together with event-averaged SWH more than 25% higher (Fig. S5c). Owing to the strong seasonality of TC activity, this high contribution is confined mainly to summer and autumn, with only limited influence in winter and spring (Fig. 3b-i; Fig. S6). Substantial TC-related contributions (20–40%) are also evident in other active coastal regions, including the Northwest Atlantic, northern Australia, the Bay of Bengal, and the eastern coasts of Madagascar (Fig. 3a).

Notably, the influence of TCs extends well beyond their tracks through long-distance swell propagation. In the equatorial coastal regions, including the central and eastern regions of Indonesia and Papua New Guinea, as well as along the northern coasts of Brazil, nearly 30% of the WSEs are generated by TCs, despite few TC tracks passing directly through the adjacent waters (Fig. S7). To more comprehensively assess the cumulative hazard potential, we therefore introduce total storm wave energy as an integrated measure of the wave-energy flux over the lifetime of each WSE [57] (see Methods; Fig. S5a). TC contributions to total energy exceed 50% in hotspot regions, and reach more than 80% along the extratropical coasts of the North Pacific, indicating a remarkably high risk of TC-induced coastal erosion.

Beyond the nearshore background-wind contribution, TCs further amplify the positive trend in WSE counts regionally. This amplification is strongest along the Northwest Atlantic (49% of the regional increase; see Methods) and partly offsets the decreasing tendency in the North Indian Ocean (Fig. 4a and b; Table S1). For both regions, the differences between the ‘TCWIND’ and ‘FILT’ trends are statistically significant at the 90% confidence level (Fig. 3j). Globally, the relative amplification is estimated at 15% (Table S1), but the difference between the two global trends is not statistically significant, reflecting the fact that TC-driven changes are concentrated in specific basins rather than uniformly distributed. These results suggest a potential future shift in wave hazard hotspots from the present-day extratropical North Pacific toward the North Atlantic, a mechanism explored in the following section.

Due to the marked decrease in TC duration across the Pacific (Fig. S8b), TCs are the primary driver (>50% local contribution in Fig. 4d) of shortening wave storm durations in key regions, such as the southwestern Mexico coasts and the Maritime Continent. This leads to a significant basin-mean trend in WSE duration of −2.8 ± 1.2%/decade in the Northeast Pacific (*P* < 0.05; Table S1). Meanwhile, although TCs also slightly modulate SWH trends, the associated changes remain marginal (<1%/decade).

Synthesizing these changes using the storm power index and total energy reveals distinct regional threats (Fig. S9). TC activity intensifies coastal wave hazards along the Yellow Sea-East China Sea region (Fig. 5) and Northwest Atlantic coasts. In contrast, rising coastal wave threats at higher latitudes (e.g. the Kuril-Aleutian region, New Zealand, and the southeastern Pacific off southern Chile and Argentina in Fig. S9c) are primarily linked to enhanced local wind stress (Fig. S4b), rather than to TCs, whose contribution is less than 10% in these regions (Fig. S9d). Remote influences of TCs also reduce wave storm hazards across the Maritime Continent while strengthening them off northeastern Brazil.

**Figure 5.**
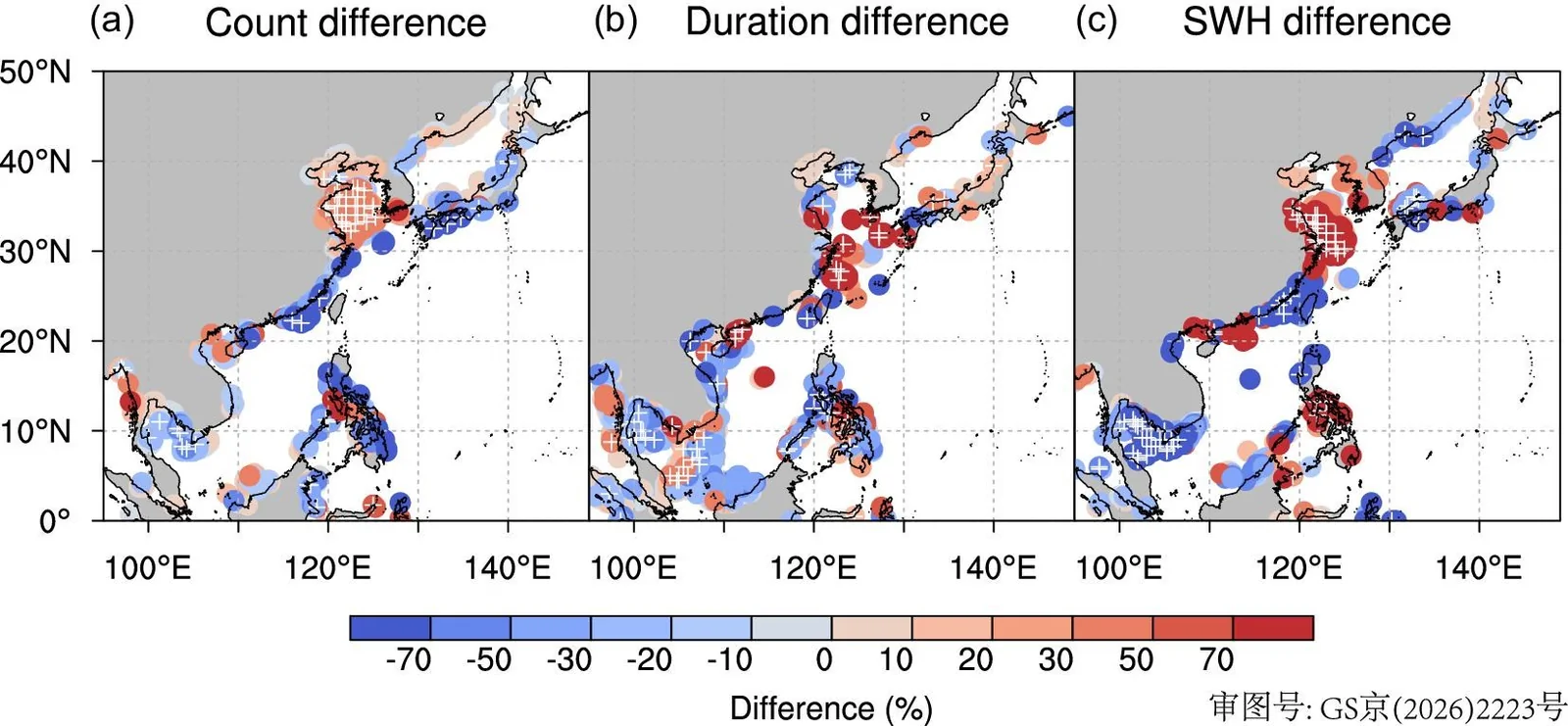
Tropical-cyclone effects on coastal wave storm trends over East Asia. Relative trend differences between the experiment ‘TCWIND’ and ‘FILT’ in wave-storm (a) count, (b) duration and (c) event-averaged SWH over the East Asian coastal region during 1990–2023, enlarged from Fig. 4b, d and f. White crosses indicate trends significant above the 95% confidence level, based on Student’s *t*-test.

### Tropical cyclone energy input explains basin-scale trend contrasts

To explain how TC activity amplifies and redistributes WSE trends across basins, we analyze the power dissipation (PD) of TCs, a metric that is used to quantify kinetic energy transfer from the atmosphere to the ocean [58–61]. Figure 6 presents the trend in annual total PD^1/3^ (see Methods), combining variability in TC count, duration and intensity (Fig. S8). Over the last 34 years (1990–2023), the global total PD^1/3^ has decreased by 6.4 ± 2.7%/decade (*P* < 0.05) relative to the 34-year mean, consistent with previous studies [62–64].

**Figure 6.**
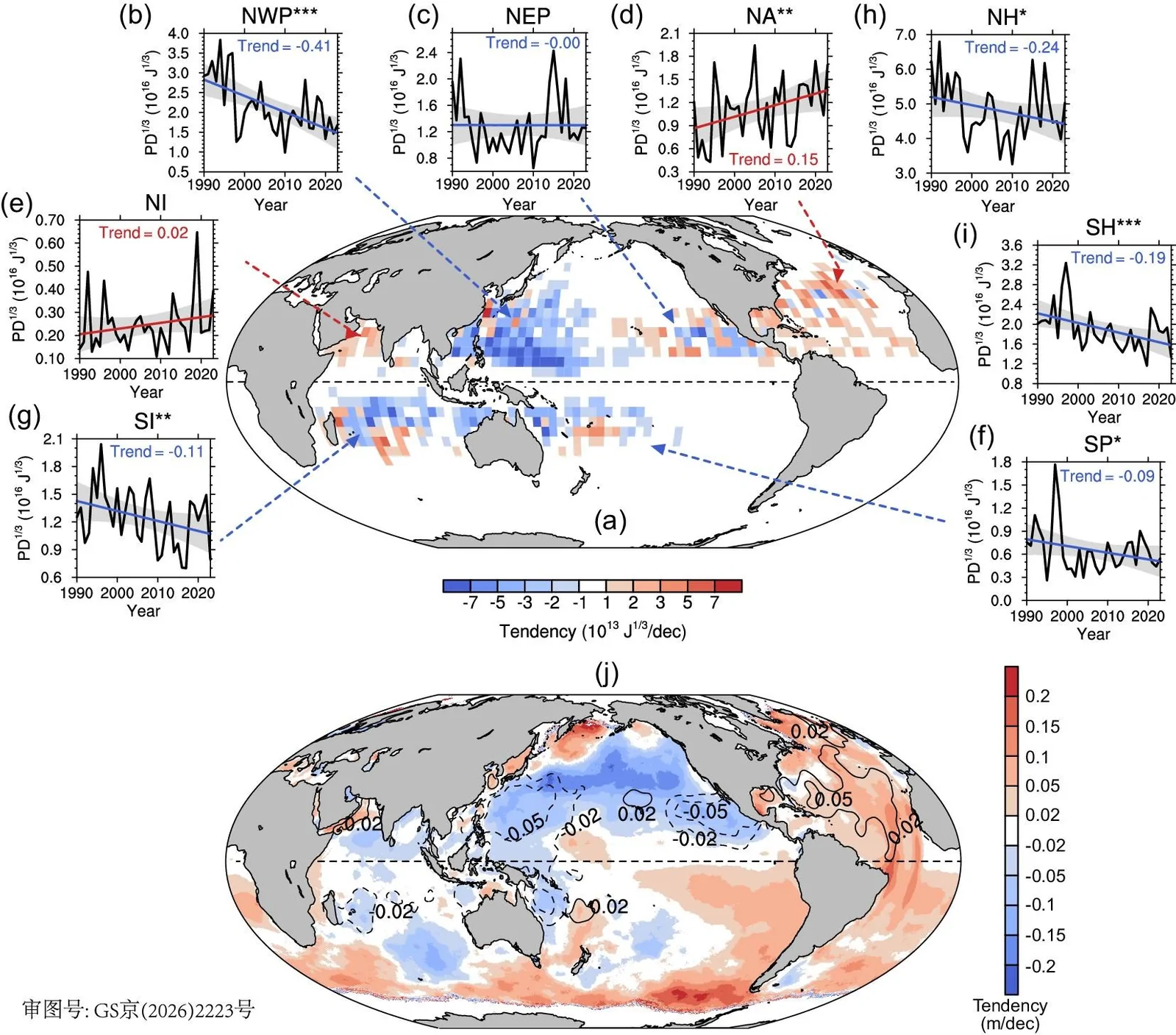
Changes in the cube root of TC-induced power dissipation (PD^1/3^) and global extreme significant wave height (SWH). (a) Global distribution of TC-induced PD^1/3^ trends (per decade) over 1990–2023 in every 4°*×*4° box; (b–i) the corresponding area-averaged time series and their linear trends in PD^1/3^; (j) global distribution of trends in the 95^th^ percentile SWH over 1990–2023 in the experiment ‘TCWIND’ (shading), together with the trend differences between the experiment ‘TCWIND’ and ‘FILT’ (contours; m/decade). NWP, NEP, NA, NI, SP, SI, NH and SH represent the Northwest Pacific, the Northeast Pacific, the North Atlantic, the North Indian Ocean, the South Pacific, the South Indian Ocean, the Northern Hemisphere and the Southern Hemisphere, respectively. One, two, and three asterisks in (b–i) indicate statistical significance at the 90%, 95%, and 99% confidence levels, respectively, based on Student’s *t*-test. Red (blue) lines denote positive (negative) linear trends (10^16^ J^1/3^/decade). Gray shading in (b–i) shows the 95% confidence interval of the linear trend.

The global decline in PD^1/3^ is largely contributed by the pronounced decrease in both TC count and duration over the Northwest Pacific (Fig. 6b; Fig. S8a and b), whereas an opposite, increasing tendency emerges in the North Atlantic, mainly due to an increase in TC count (Fig. 6d; Fig. S8a). Under global warming, sea surface temperatures have exhibited a La Niña-like pattern [65], characterized by cooling in the tropical eastern Pacific and relative warming elsewhere. This pattern strengthens the Walker circulation [65], and then weakens upper-level westerly anomalies and vertical wind shear over the tropical North Atlantic, thereby favoring the growth of disturbance activity there [4,66]. In contrast, over the Northwest Pacific, the strengthened zonal sea surface temperature gradient shifts convective activity toward the Maritime Continent region, leaving the main TC genesis region with a weakened monsoon trough, reduced low-level cyclonic shear, anomalous subsidence and mid-tropospheric drying [67,68]. These changes intensify disturbance activity over the North Atlantic but weaken it over the Northwest Pacific, and by modulating the seeds of TCs, they further drive more frequent and clustered TCs in the North Atlantic while suppressing TC genesis in the Northwest Pacific (Fig. S8a) [32]. These basin-scale contrasts in TC activity ultimately lead to a shift of coastal wave storm hotspots toward the North Atlantic.

Regionally, PD^1/3^ trends help explain the observed WSE patterns. The significant PD^1/3^ increase in the North Atlantic (13.5 ± 6.1%/decade; Fig. 6d) physically underpins the amplified rise in extreme SWH (Fig. 6j), and consequently, the increase in coastal WSE count there. Although the North Indian Ocean exhibits a comparable PD^1/3^ increase of 10.0 ± 8.0%/decade, mainly associated with enhanced TC intensity there (Fig. S8c), this trend is not statistically significant because it is spatially confined, with notable enhancements primarily restricted to the Arabian Sea coastal regions (Fig. 6a and e). These regional PD^1/3^ patterns help explain both the mitigation of the WSE count decrease in the North Indian Ocean when TC effects are included (Fig. 3j) and the enhanced remote responses along the northeastern coasts of Brazil.

Conversely, significant PD^1/3^ decreases are evident in the Northwest Pacific (−19.0 ± 5.0%/decade; Fig. 6b), the South Pacific (−13.4 ± 7.9%/decade; Fig. 6f) and the South Indian Ocean (−8.7 ± 4.3%/decade; Fig. 6g). These decreases cause the negative trend in extreme SWH (Fig. 6j), which is otherwise mainly confined to the mid- to high-latitude North Pacific, to extend into TC hotspot regions of the Northwest and Northeast Pacific, as well as the Maritime Continent, ultimately leading to concurrent reductions in WSE duration and intensity across the Maritime Continent. Embedded within these widespread declines, however, the Yellow Sea-East China Sea region exhibits a marked PD^1/3^ enhancement of 28.4 ± 12.8%/decade, corresponding to an absolute trend of 7.28*×*10^13^ J^1/3^/decade normalized by the regional 34-year mean of 2.57*×*10^14^ J^1/3^. Over the same region, the trend difference in wave storm count between the ‘TCWIND’ and ‘FILT’ experiments is also positive and statistically significant at the 95% confidence level (Fig. 5a). This localized increase is likely driven by the poleward migration of TCs toward the coasts [1], highlighting a localized intensification of coastal wave hazards.

## DISCUSSION

Our study provides a systematic global quantification of long-term trends of coastal extreme wave events and their driving wind environment, with TCs treated as an amplifier embedded within the background wind forcing. A key finding is the significant global increase in coastal wave storm occurrence frequency (1.9 ± 0.8% per decade), a trend primarily driven by enhanced background wind stress. TCs further modulate this wind-driven signal regionally by increasing the summer and autumn shares of WSE occurrence in TC-prone regions, accounting for 49% of the increase along the Northwest Atlantic coasts, and partly offsetting the decreasing tendency in the North Indian Ocean. The Yellow Sea-East China Sea region stands out as a hotspot where TC-driven amplification of wave storm frequency is statistically significant at the 95% confidence level (Fig. 5), consistent with a marked increase in TC power dissipation. This highlights a compound influence in which large-scale climate changes set the basin-wide trend, while TC activity reshapes coastal hazards across key regions. Conversely, we find that variability in TC activity is the principal driver behind a pronounced shortening of wave storm duration across the Maritime Continent (about 4–6 hours per decade), a counterintuitive trend linked to significant declines in TC-induced power dissipation in the Pacific basins.

As a composite metric that integrates TC intensity, frequency, duration, and translation speed, PD^1/3^ serves as a robust proxy for TC energy input to the ocean. Its temporal variations are strongly consistent with those of WSEs, supporting its use for interpreting wave storm behavior. Nevertheless, the trends in PD^1/3^ are much larger than the rates of change in WSE-related factors, because PD^1/3^ captures not only the energy transferred to surface waves, but also the portions transferred to ocean currents and turbulence [69–71]. Moreover, not all TC-generated waves necessarily propagate shoreward to contribute to coastal WSEs.

To date, no globally consistent WSE dataset is available for storm-dominated coastal regions, owing to both uncertainties in global wave data and inconsistencies in storm identification methods. In this study, the limited model resolution and the idealized structure of the corrected TC wind fields inevitably introduce uncertainties into WSE identification, especially for event duration and wave height in narrow coastal regions. Validation against over 25 years of buoy records shows that the ‘TCWIND’ experiment underestimates the annual mean event count by approximately 9.8% (or −0.9 events), a bias within an acceptable range. This underestimation suggests that the actual storm-induced coastal hazards may be even more severe than those represented in our simulations.

Finally, as global climate models continue to evolve toward finer resolutions and more realistic air-sea-wave coupling, it becomes increasingly important to quantify and understand compound hazards associated with TCs. Our analysis identifies the Northwest Atlantic coasts as hotspot regions, where TC activity is driving the strongest escalation of coastal wave-hazard risk. In contrast, although the Northwest Pacific as a whole shows a decreasing threat, the Yellow Sea-East China Sea region emerges as an embedded subregion with a pronounced rise in TC-driven wave hazards (Fig. 5). These findings gain heightened significance amid conflicting climate projections. While 22 of 27 recent studies project reduced global TC frequency under global warming [72], an intensification of TCs is consistently anticipated [11,73,74]. Furthermore, the observed increase in TC clustering in the North Atlantic has already amplified flood hazards along the eastern coast of North America [5,32,34]. Together, these factors create substantial uncertainty regarding future coastal risks in these identified hotspots. Regardless of this uncertainty, our results underscore the urgent need to strengthen monitoring, predictive capacity, and adaptive preparedness for TC-driven wave hazards in vulnerable coastal regions.

## METHODS

### Data

The 6-h global TC tracks and winds from 1990 to 2023 are taken from the International Best Track Archive for Climate Stewardship Project (IBTrACS, Version 4r01) [51]. The analysis is restricted to 1990–2023 because pre-1990 TC records contain substantial uncertainties in intensity and position, arising from changes in observing systems and agency-specific analysis practices. To drive the wave model, we employ 3-h 10-m wind vectors from the Japanese 55-year Reanalysis based surface dataset for forcing ocean-sea ice models (JRA55-do) at a horizontal resolution of about 50 km [50]. JRA55-do is a bias-corrected dataset derived from JRA-55 for ocean simulations, with surface meteorological fields adjusted against observational and multi-reanalysis reference products, including corrections to surface wind fields around TCs [75]. Compared with daily observations from Advanced Scatterometer (ASCAT) onboard Meteorological Operational satellite A [76], JRA55-do shows reduced negative biases in 10-m wind speeds relative to ERA5 (Fig. S10), particularly over the Yellow Sea-East China Sea and Kuroshio regions. By contrast, ERA5 and JRA55-do exhibit similar spatial patterns of wind-direction bias (Fig. S11). The ERA5 reanalysis [77] is also used to provide sea-ice concentration for defining the ice mask in the model. The global remote sensing daily merged multi-mission along-track SWH L3 products (1992–2018) [44] and SWH observations from available National Data Buoy Center buoys [78] are applied to validate the simulations, with model outputs interpolated to the observation locations. In addition, the Earth Topography 2022 global seamless topographic-bathymetric dataset [79] is used to identify the coastal areas, with water depths shallower than 200 m.

### Model

The WAVEWATCH III version 5.16 model [80] is used to simulate the global ocean surface waves. The model is configured with a spatial resolution of 1/4° and the latitude range is 90°S-90°N. The spectral space is discretized into 36 directions with a 10° resolution and 50 frequencies ranging from 0.035 to 0.964 Hz, and the high frequency tail assumption is used with a spectral level decaying as *f*  ^−5^ (frequency). For the input-dissipation source term, the ST4 package [81] is chosen, and the parameters used for ST4 follow TEST471f [80,82]. The maximum global time step is set to 900 s, with time steps of 300 s for spatial propagation, 450 s for refraction, and a minimum of 30 s for source terms. Given the complexity of wave-ice interactions, which are beyond the scope of this study, the related processes are turned off, and the analysis is restricted to 60°S–60°N.

### Experiments

The WAVEWATCH III model is driven by 10-m wind forcing modified from the JRA55-do reanalysis, which incorporates bias corrections based on satellite observations and multiple atmospheric reanalysis products to improve surface forcing for ocean-sea ice simulations and to more accurately represent TC characteristics [10,50]. However, owing to the relatively coarse resolution and limitations in physical parameterizations, global reanalysis products tend to severely underestimate TC intensity, often by more than 30%, particularly for those intense, compact, and fast-moving ones [46–49]. Therefore, a parametric cyclone model is required to be incorporated into the reanalysis winds, to provide more realistic wind fields both near and far from TCs [10,27,83,84].

Following Vincent *et al.* [84] and Zhang *et al.* [10], the TC signals in the JRA55-do wind fields are first filtered out to produce the JRA55-do-FILT data, after which an idealized TC vortex is embedded to generate the JRA55-do-TC data. Based on the TC positions and intensities from IBTrACS and 10-m winds from JRA55-do interpolated to hourly, an 11-day running mean is applied within 600 km of each TC center, followed by a linear transition to the original winds from 600 to 1200 km:


(1) \begin{eqnarray*}
{U}_{\textit{FILT}} = \omega {U}_{{\rm mean}} + \left( {1 - \omega } \right){U}_{{\mathrm{\ }}{\rm original}},
\end{eqnarray*}

(2) \begin{eqnarray*}
\omega = \left\{ {\begin{array}{@{}c@{\quad}c@{\quad}c@{}}
1, & r \le 600\\
\left( {1200 - r} \right)/600, & 600 < r \le 1200\\
0, & r > 1200 \end{array}} \right.\!\!\!\!\! .
\end{eqnarray*}

Here, *r* denotes the distance from each grid point to the TC center. The above process yields the JRA55-do-FILT data, and then the idealized ${U}_{\textit{vortex}}$ pattern is embedded into ${U}_{{\rm FILT}}$, to generate the JRA55-do-TC data:


(3) \begin{eqnarray*}
{U}_{TC} = \omega {U}_{{\rm vortex}} + {U}_{{\rm FILT}},
\end{eqnarray*}

where ${U}_{{\rm vortex}}$ is derived from statistical fitting to observed TC wind profiles [85]. This method alleviates the negative bias of TC intensity in JRA55-do and restores a more realistic and compact TC eyewall [10].

Two numerical experiments (referred to as ‘TCWIND’ and ‘FILT’) are conducted to evaluate the effects of TC on coastal extreme wave events, driven by the 1-h wind forcing of JRA55-do-TC and JRA55-do-FILT data, respectively. The difference between the ‘TCWIND’ and ‘FILT’ experiments is used to isolate the net accumulative influence of TCs on waves, whereas the ‘FILT’ experiment alone represents the effects of non-TC events. Both experiments are run for 34 years (1990–2023), with initial wave fields generated from 10-day spin-up runs starting from rest. Similarly, another additional sensitivity experiment ‘JRA’, is conducted using the original JRA55-do winds as forcing, to assess the impact of TC wind correction.

### Wave storm definition

Following previous studies [12–17], the wave storms are defined using the local 95th percentile of historical SWH. This relative threshold is appropriate for a global climatological assessment, whereas absolute wave-height thresholds, more commonly used in regional studies, are also important for capturing local hazard relevance in terms of societal damage, infrastructure risk and adaptation planning. An event of wave storm is recorded when SWH exceeds the local 95th percentile for a minimum of 12 hours. Besides, two consecutive events are treated independently only if the time interval between their peak SWH exceeds 48 hours, otherwise they are merged into a single event.

The main parameters used include the count, duration and mean SWH of events, as well as two composite metrics, the storm power index (SPI) and the total storm wave energy (TSWE):


(4) \begin{eqnarray*}
SPI = {H}_s^2 \cdot {T}_d,
\end{eqnarray*}

(5) \begin{eqnarray*}
\textit{TSWE} = \int {P}_wdt.
\end{eqnarray*}

Here, ${H}_s$ is the mean SWH within an event, and ${T}_d$ denotes its corresponding duration; total energy is obtained by integrating the wave energy flux (${P}_w = {C}_gE$) over the lifespan of a wave storm event, where ${P}_w$ is determined by the wave group velocity ${C}_g$ and wave energy density $E = {({H}_s/4)}^2$. Only the WSEs occurring in coastal areas are considered in this study, with water depths shallower than 200 m.

### TC-induced power dissipation

The TC-induced PD [58] is calculated as


(6) \begin{eqnarray*}
PD{\mathrm{\ }} = \int\!\!\!\int {\rho }_{air}{C}_d{U}_{10}^3\textit{dAdt}.
\end{eqnarray*}

(7) \begin{eqnarray*}
{C}_d{\mathrm{\ }} = \left\{ {\begin{array}{@{}*{1}{c}@{}} {\frac{{{a}_1}}{{{U}_{10}}} + {a}_2 + {a}_3{U}_{10} + {a}_8{U}_{10}^6,\ ({U}_{10} < 33\, {\rm m/s})}\\ {0.00234,\ \left( {{U}_{10} \ge 33\, {\rm m/s}} \right)\ } \end{array}} \right..
\end{eqnarray*}

Here, ${\rho }_{air}$ = 1.29 kg/m^3^ is the surface air density, ${U}_{10}$ is the 10-m wind speed, ${C}_d$ is the surface momentum exchange coefficient parameterized following Large and Yeager [86]; ${a}_1$, ${a}_2$, ${a}_3$ and ${a}_8$ are constant coefficients; *A* is the 7°×7° domain centered on the TC center [60,61], and *t* is the TC lifespan. The cube root of PD (PD^1/3^) is used as a more physically representative metric of TC-induced oceanic energy input, compared with PD itself [59,62]. It is integrated over each TC’s lifespan and summed across all TCs within a year, to obtain the annual total PD^1/3^.

### Standard error of linear trend

The standard error of linear trend [87] is calculated as


(8) \begin{eqnarray*}
SE = \sqrt {\frac{{\mathop \sum \nolimits_{i = 1}^n {{\left( {{y}_i - {{\hat{y}}}_i} \right)}}^2}}{{\left( {n - 2} \right)\mathop \sum \nolimits_{i = 1}^n {{\left( {{x}_i - \bar{x}} \right)}}^2}}} .
\end{eqnarray*}

Here ${y}_i$ denotes the value of the variable used for trend estimation in year i (i = 1, 2, …, 34), ${\hat{y}}_i$ denotes the fitted value from the ordinary least-squares linear regression, ${x}_i$=1990, 1991, …, 2023, $\bar{x}$ is the mean of ${x}_i$, and n = 34 is the number of years used to estimate the linear trend. In Fig. 3j, overlapping error bars cannot be used to assess the significance of differences between the paired trends, because the ‘TCWIND’ and ‘FILT’ trends are not independent and share substantial year-to-year covariance. Therefore, we assess the trend differences using linear regression of the annual ‘TCWIND’ minus ‘FILT’ difference series. For the North Indian Ocean and the Northwest Atlantic, where the trend differences are significant, the Gaussian error assumption is reasonable based on the residual diagnostics in Fig. S12.

### Relative trend amplification

The relative trend amplification (%) is calculated as


(9) \begin{eqnarray*}
RA = \frac{{{t}_{{\rm TCWIND}} - {t}_{{\rm FILT}}}}{{{t}_{{\rm FILT}}}} \times 100{\mathrm{\% }}.
\end{eqnarray*}

Here ${t}_{{\rm TCWIND}}$ and ${t}_{{\rm FILT}}$ denote the linear trends in the ‘TCWIND’ and ‘FILT’ experiments, respectively.

## Supplementary Material

nwag528_Supplemental_File

## Data Availability

The International Best Track Archive for Climate Stewardship Project Version 4r01 data can be found in the NOAA National Centers for Environmental Information (https://www.ncei.noaa.gov/products/international-best-track-archive). The Earth Topography 2022 data can also be downloaded from the NOAA National Centers for Environmental Information (https://www.ncei.noaa.gov/products/etopo-global-relief-model). The National Data Buoy Center buoy data is obtained from NOAA (https://www.ndbc.noaa.gov). The JRA55-do data can be found under the website of Earth System Grid Federation (https://aims2.llnl.gov/search/input4mips). The Global remote sensing daily merged multi-mission along-track SWH, L3 product, is available at the Centre for Environmental Data Analysis (http://catalogue.ceda.ac.uk/uuid/3ef6a5a66e9947d39b356251909dc12b). The fifth generation European Centre for Medium-Range Weather Forecasts Reanalysis (ERA5) data is available at the Copernicus Climate Change Service Climate Data Store (https://cds.climate.copernicus.eu/datasets/reanalysis-era5-single-levels?tab=download). The ASCAT data can be downloaded from the Remote Sensing Systems (https://data.remss.com/).
