# Supplementary material for: Rising coastal wave hazards from climate-driven winds and tropical cyclones
Source: Natl Sci Rev. 2026 Aug 22;13(18):nwag528. doi: 10.1093/nsr/nwag528 (PMC13639191; doi:10.1093/nsr/nwag528)
Supplement: Supplementary file 1 [file nwag528_supplemental_file.pdf]

Supplementary Materials for

Rising Coastal Wave Hazards from Climate-Driven Winds and Tropical Cyclones

Ruizi Shi *et al.*

*Corresponding author. Email: fxu@mail.tsinghua.edu.cn

This PDF file includes:

Figs. S1 to S12
Table S1

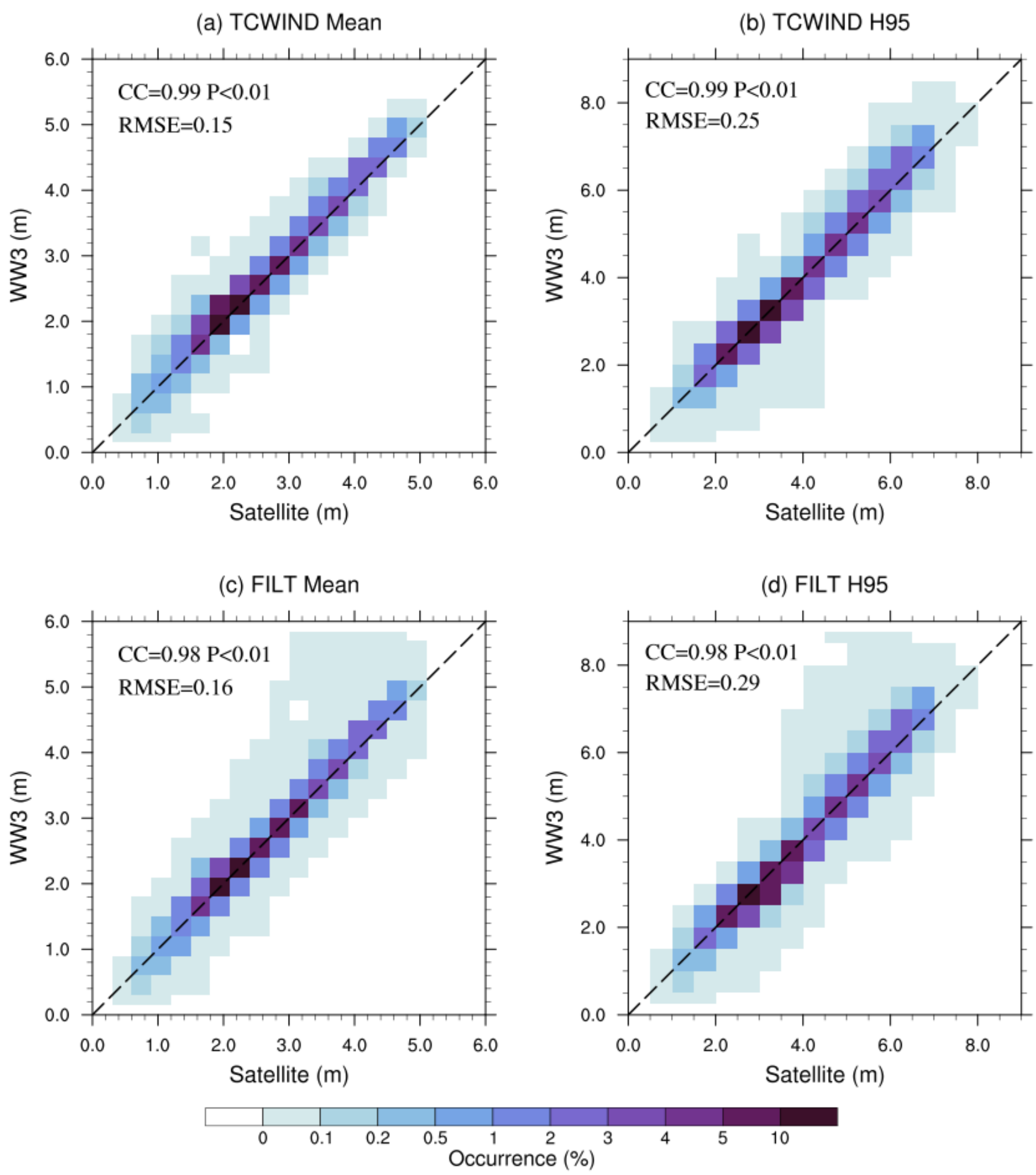

Fig. S1.

The occurrence frequency in 2-dimensional space of satellite results and experiment results for (**a**) annual mean SWH and (**b**) 95th percentile SWH in the experiment "TCWIND", as well as (**c**) annual mean SWH and (**d**) 95th percentile SWH in the experiment "FILT". The text in the upper left corner denotes the correlation coefficient and corresponding p-value, as well as the root mean square error.

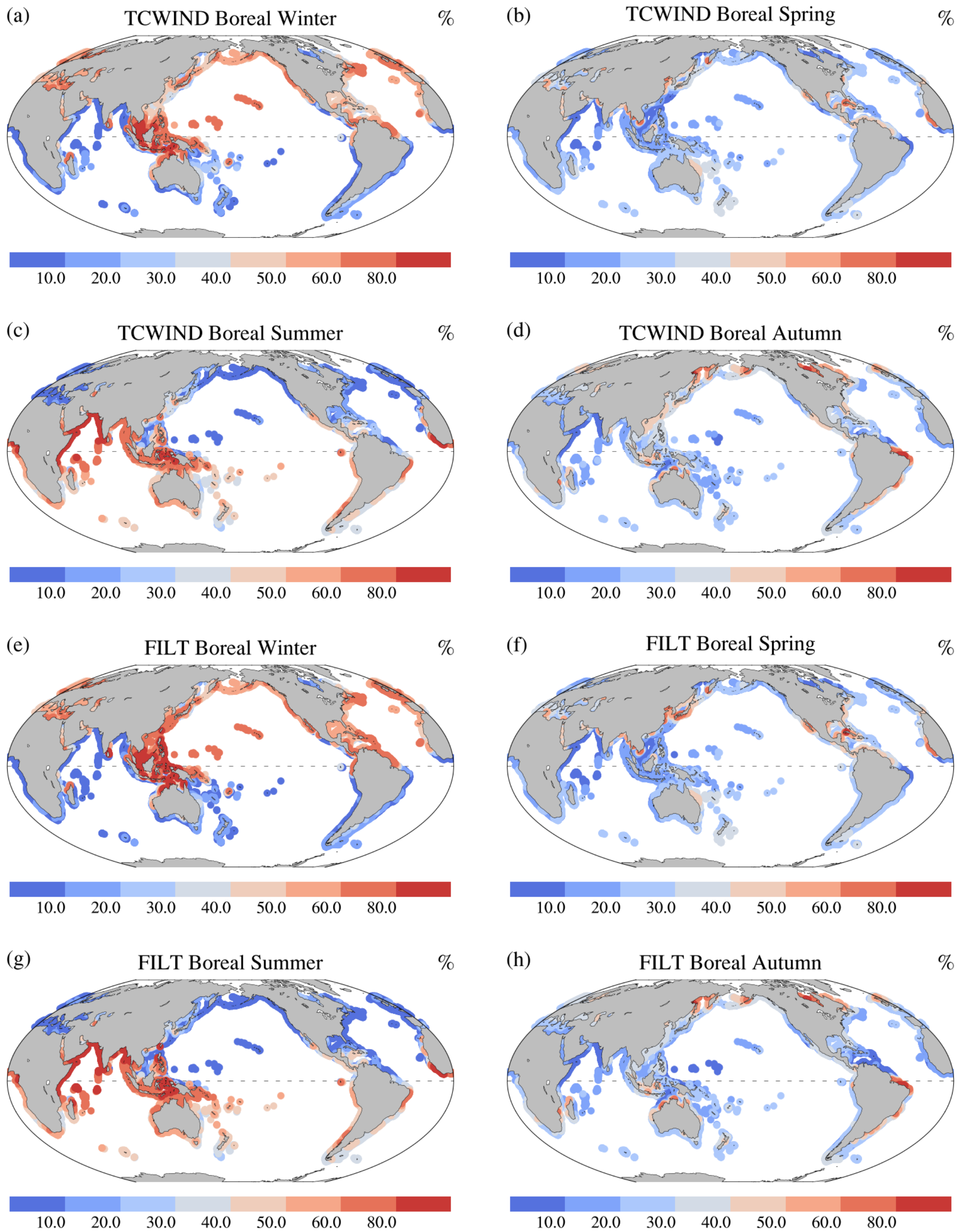

Fig. S2.

Seasonal distribution of coastal wave storm occurrences in “TCWIND” and “FILT” experiments: (**a&e**) December-February, (**b&f**) March-May, (**c&g**) June-August and (**d&h**) September-November. Percentages are expressed relative to the local annual total counts.

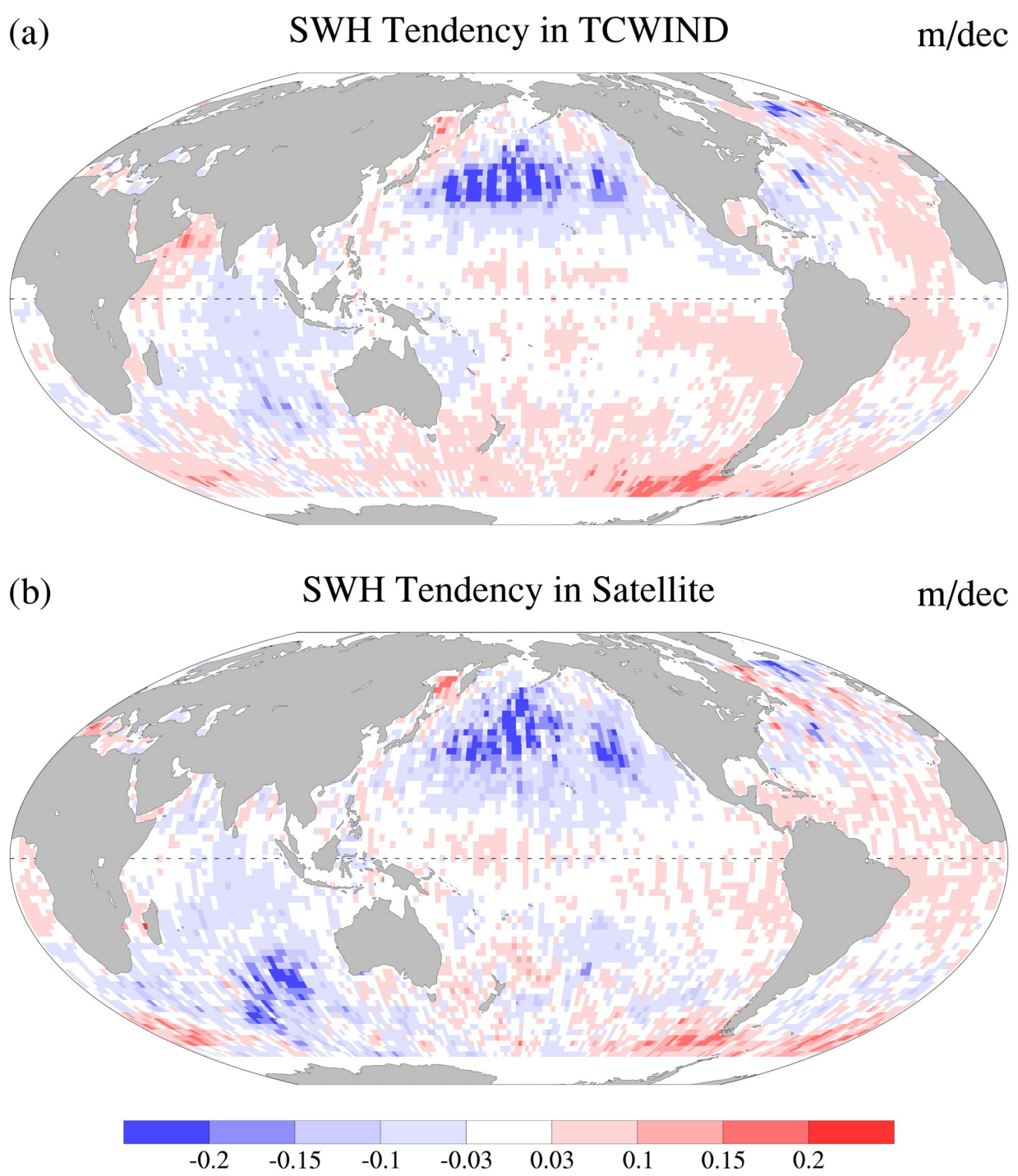

Fig. S3.

Linear trends in the annual 95th percentile of SWH for (**a**) “TCWIND” experiment and (**b**) satellite observation. The model outputs are interpolated to the along-track observation locations.

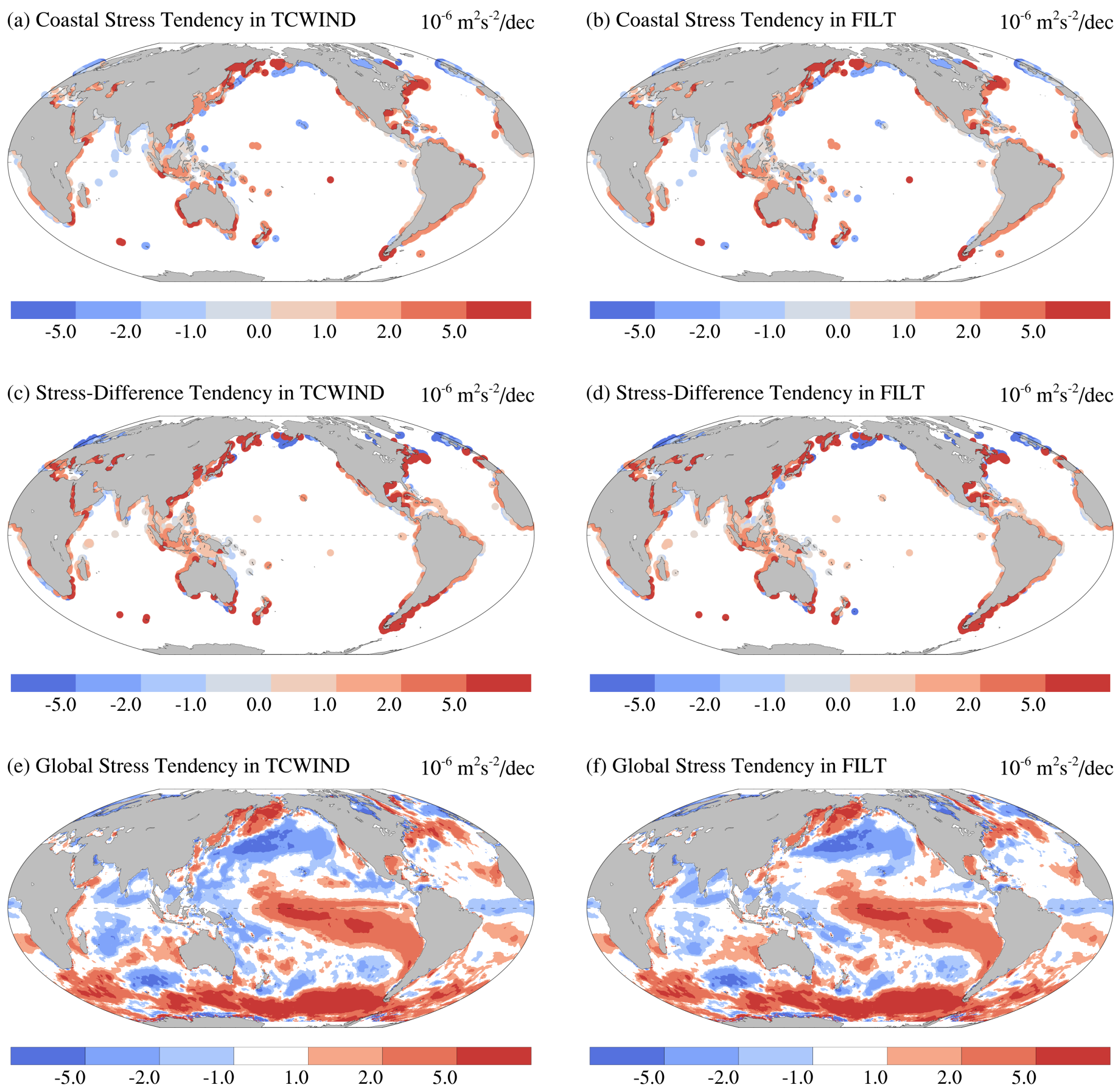

Fig. S4.

Linear trends in annual mean coastal wind stress in the (**a**) "TCWIND" and (**b**) "FILT" experiments, and linear trends in the annual 95th percentile of the difference between daily maximum and daily mean coastal wind stress in the (**c**) "TCWIND" and (**d**) "FILT" experiments. Linear trends in annual mean global wind stress are shown for the (**e**) "TCWIND" and (**f**) "FILT" experiments. Only statistically significant trends above the 90% confidence level are shown in (a)-(d), based on Student's t-test.

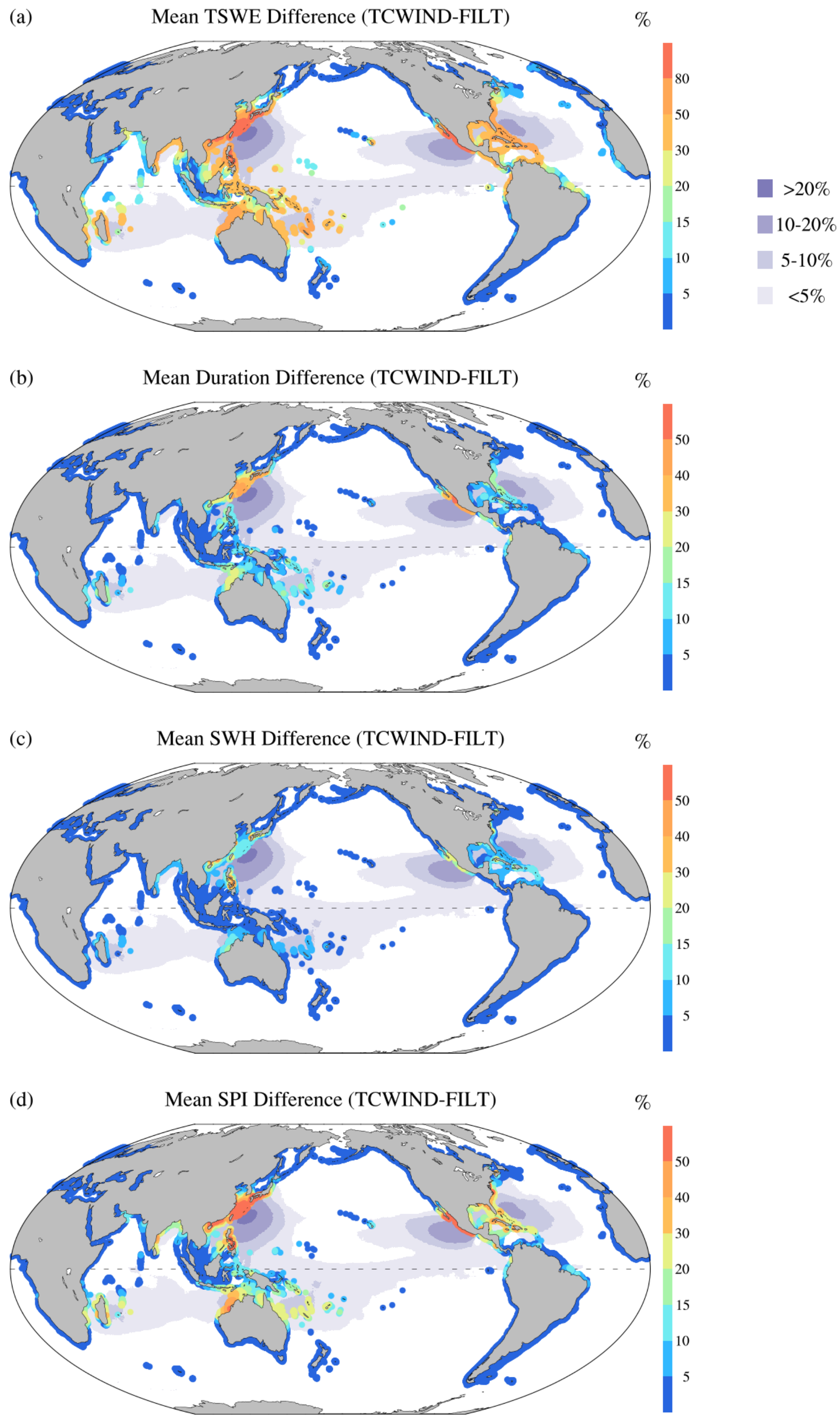

Fig. S5.

Global coastal annual mean of relative difference (%) between the experiment "TCWIND" and "FILT" in the (**a**) total storm wave energy (TSWE), (**b**) duration, (**c**) event-averaged SWH and (**d**) storm power index (SPI) of wave storm events over 1990-2023. Percentages are defined as the difference between "TCWIND" and "FILT", expressed relative to the "TCWIND" experiment. The corresponding relative difference of 95th percentile SWH is indicated with purple shading, expressed relative to "TCWIND".

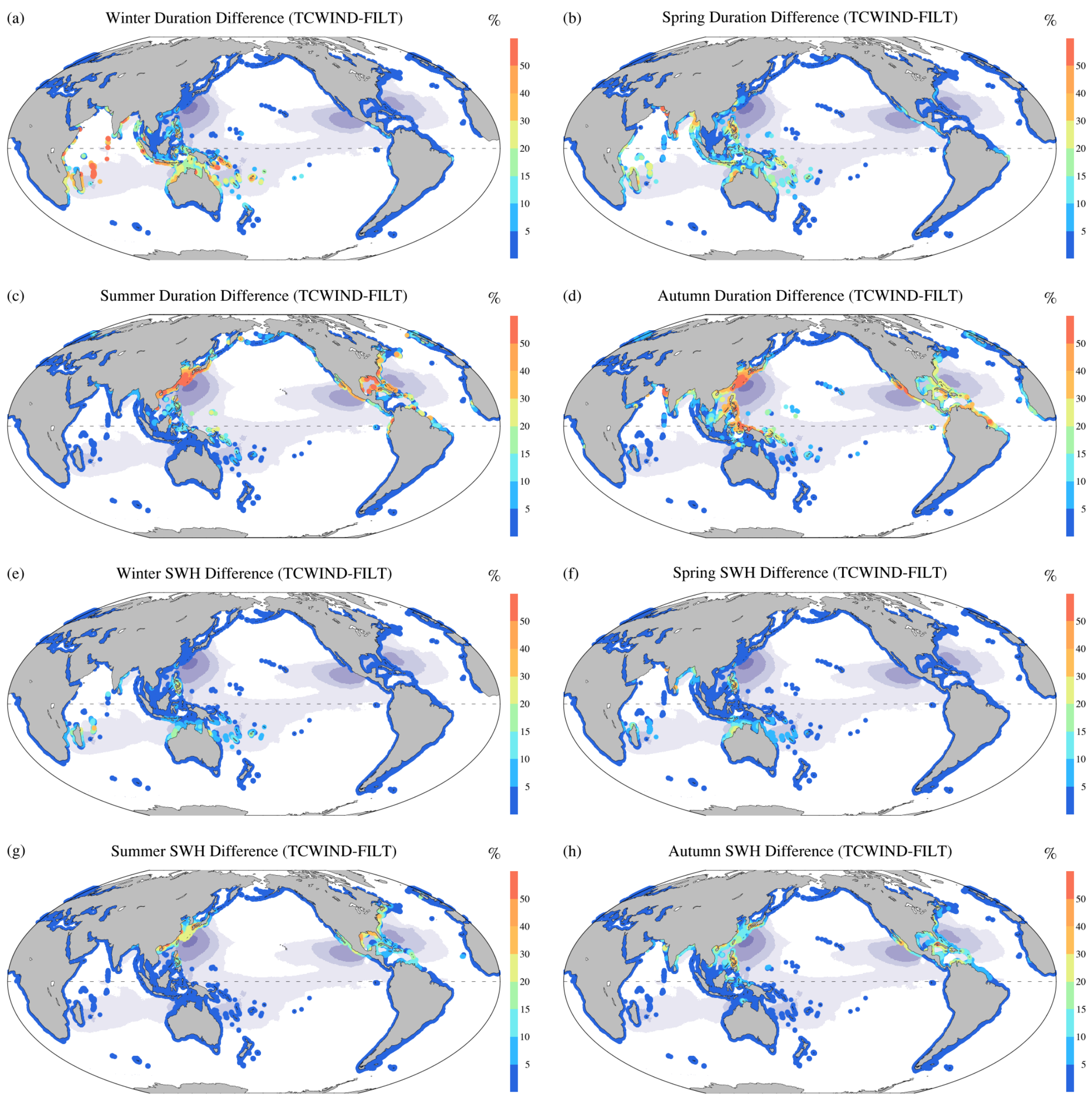

Fig. S6.

Global coastal seasonal mean of relative difference (%) between the experiment "TCWIND" and "FILT" in (**a-d**) duration and (**e-h**) event-averaged SWH for (**a&e**) winter, (**b&f**) spring, (**c&g**) summer and (**d&h**) autumn. Percentages are defined as the difference between "TCWIND" and "FILT", expressed relative to the "TCWIND" experiment. The corresponding relative difference of 95th percentile SWH annual mean is indicated with purple shading, expressed relative to "TCWIND".

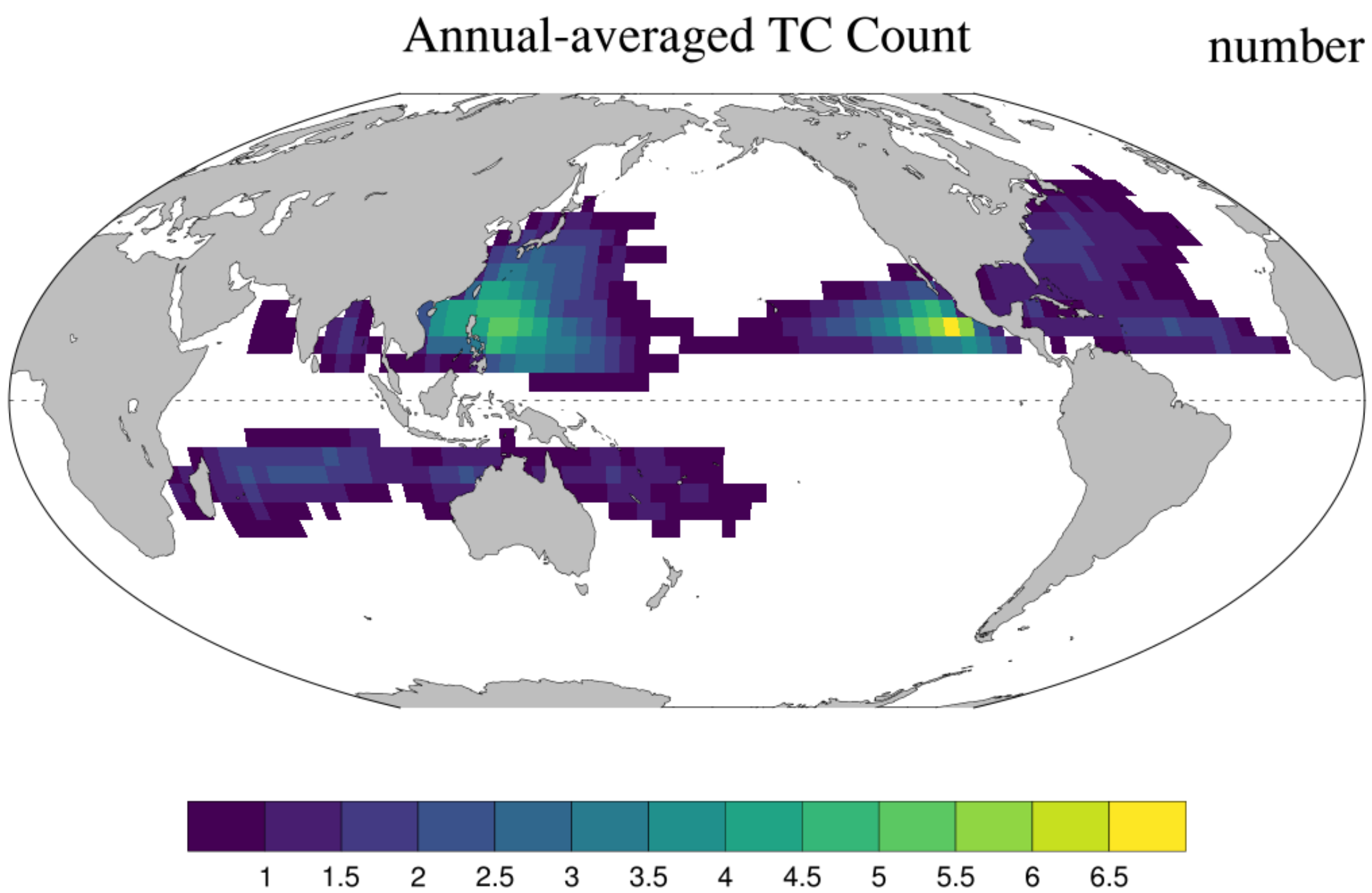

Fig. S7.

The annual-averaged distribution of the TC count in every 4°×4° box

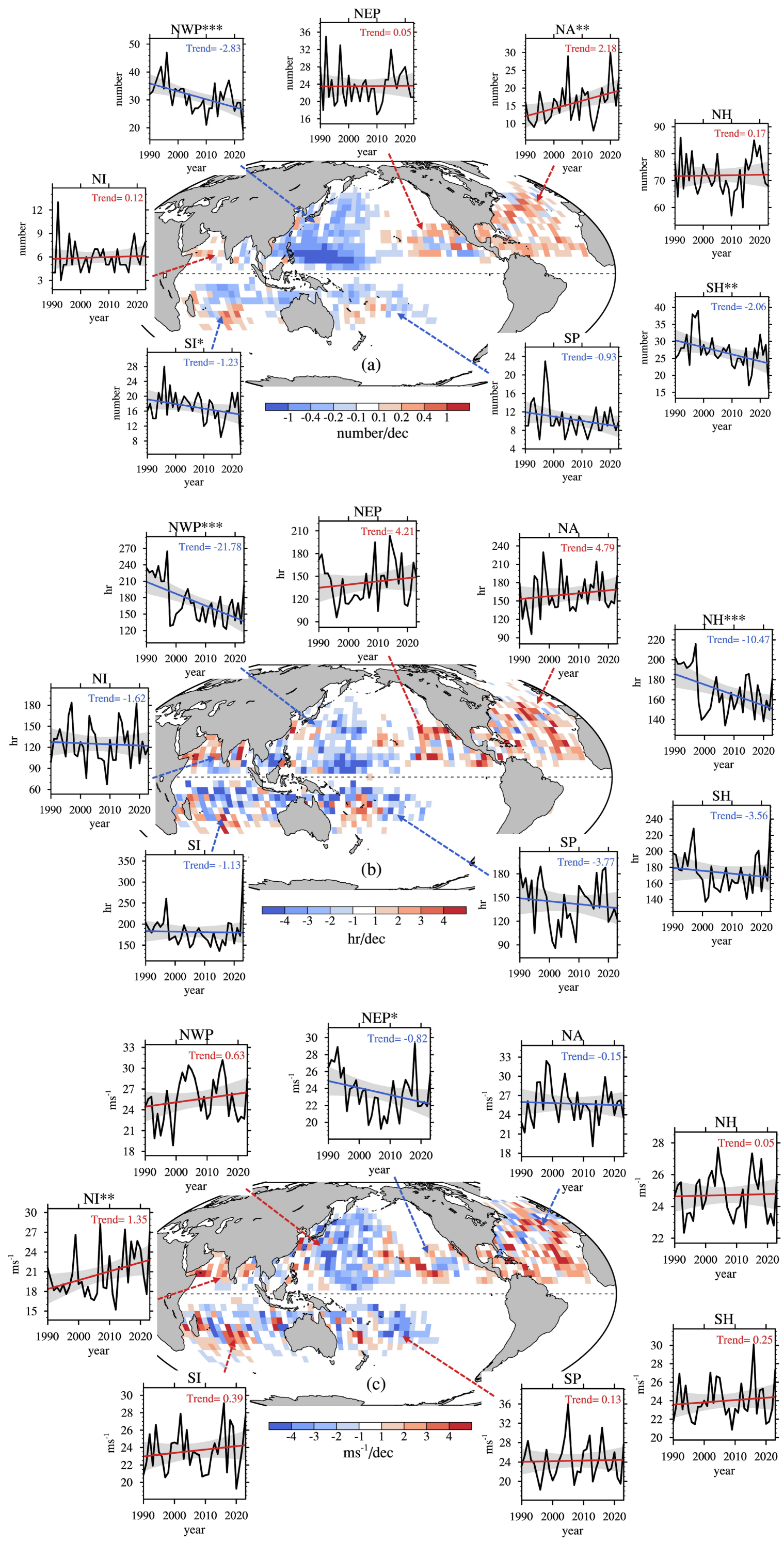

Fig. S8.

Trends in TC characteristics. The global distributions are trends (per decade) of (**a**) TC count, (**b**) TC duration and (**c**) TC intensity over 1990-2023 in every 4°×4° box. The corresponding area-averaged time series and their linear trends are shown in the surrounding panels. One, two, and three asterisks indicate statistical significance at the 90%, 95%, and 99% confidence levels, respectively, based on Student's t-test. Red (blue) lines denote positive (negative) linear trends. Gray shading shows the 95% confidence interval of the linear trend.

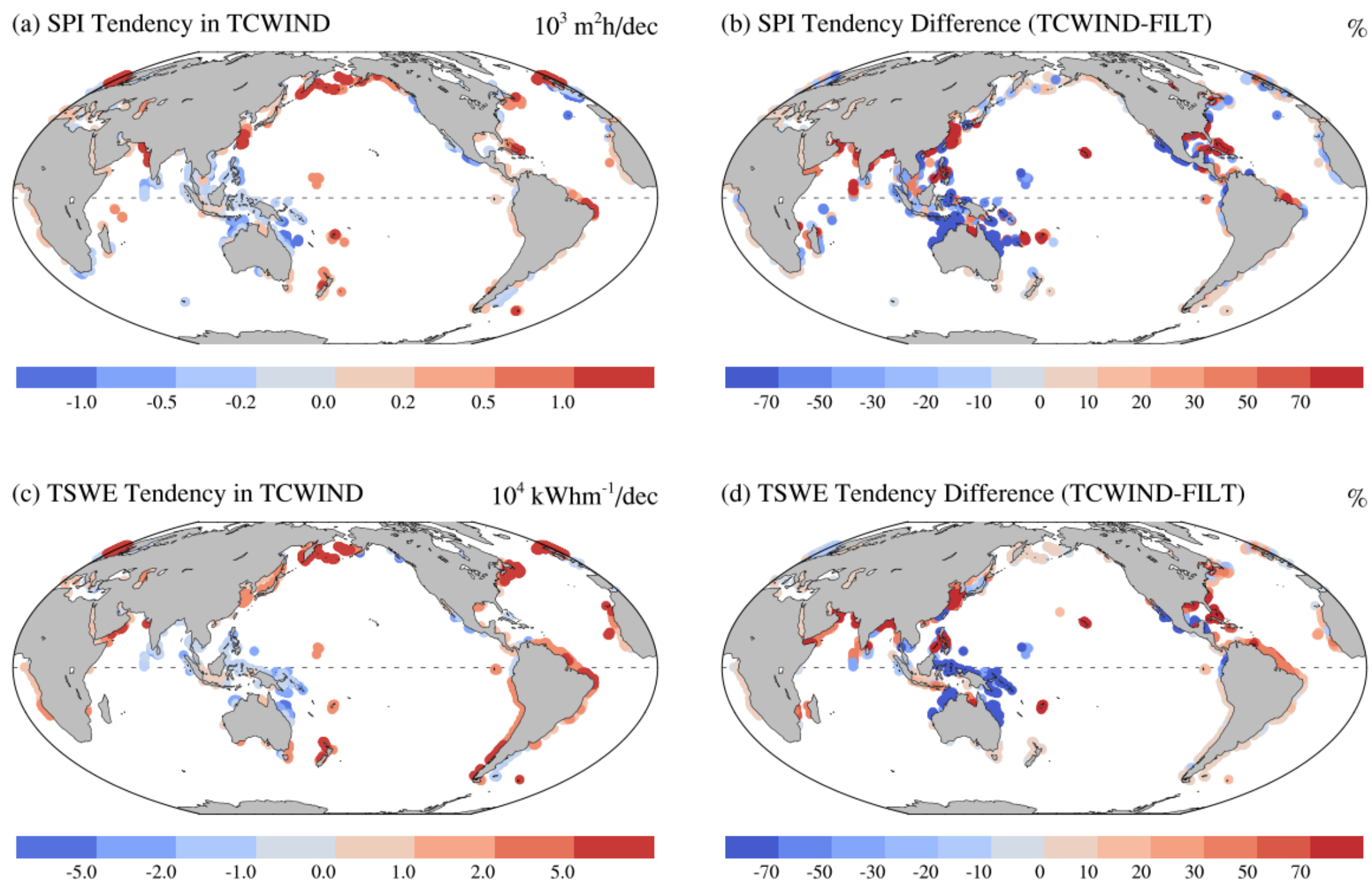

Fig. S9.

Linear trends (per decade) of the (**a**) storm power index (SPI), and (**c**) total storm wave energy (TSWE) of wave storms in global coastal areas for the experiment "TCWIND", over 1990-2023; (**b&d**) represent the corresponding relative trend differences (%) between the experiment "TCWIND" and "FILT". Percentages are defined as the differences between "TCWIND" and "FILT", expressed relative to the absolute values of the "TCWIND" trends. Only statistically significant trends above the 90% confidence level are shown, based on Student's t-test.

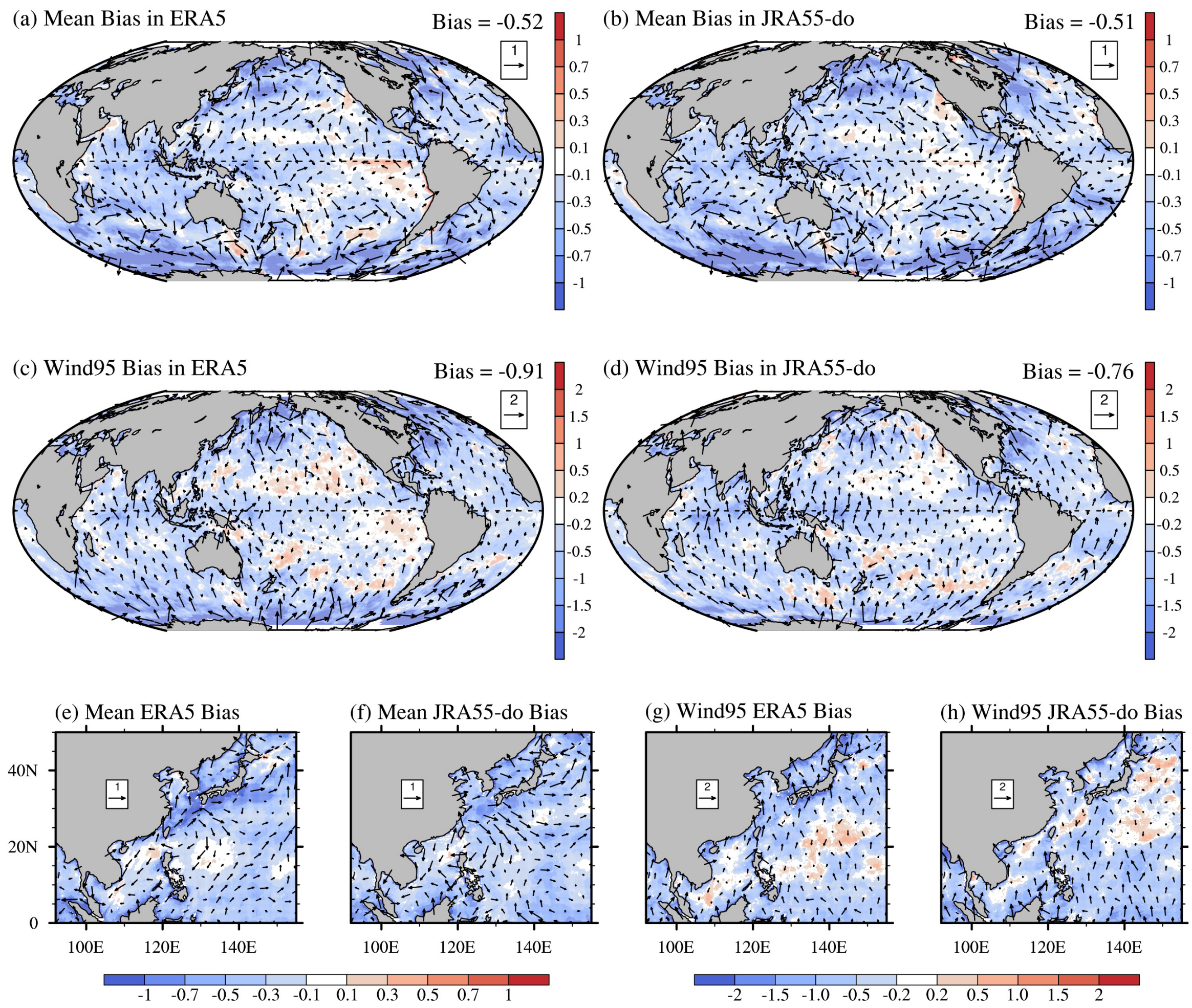

Fig. S10.

Biases of ERA5 and JRA55-do reanalysis in the (**a&b**) annual mean and (**c&d**) 95th-percentile 10-m winds (m/s) relative to ASCAT during 2008-2020, with the corresponding enlarged views over East Asia shown in (**e-h**). Poor-quality ASCAT records affected by rain are removed. The reanalysis winds are interpolated to the along-track ASCAT locations. Shading represents wind speed bias, calculated as reanalysis minus ASCAT, and vector represents wind vector bias.

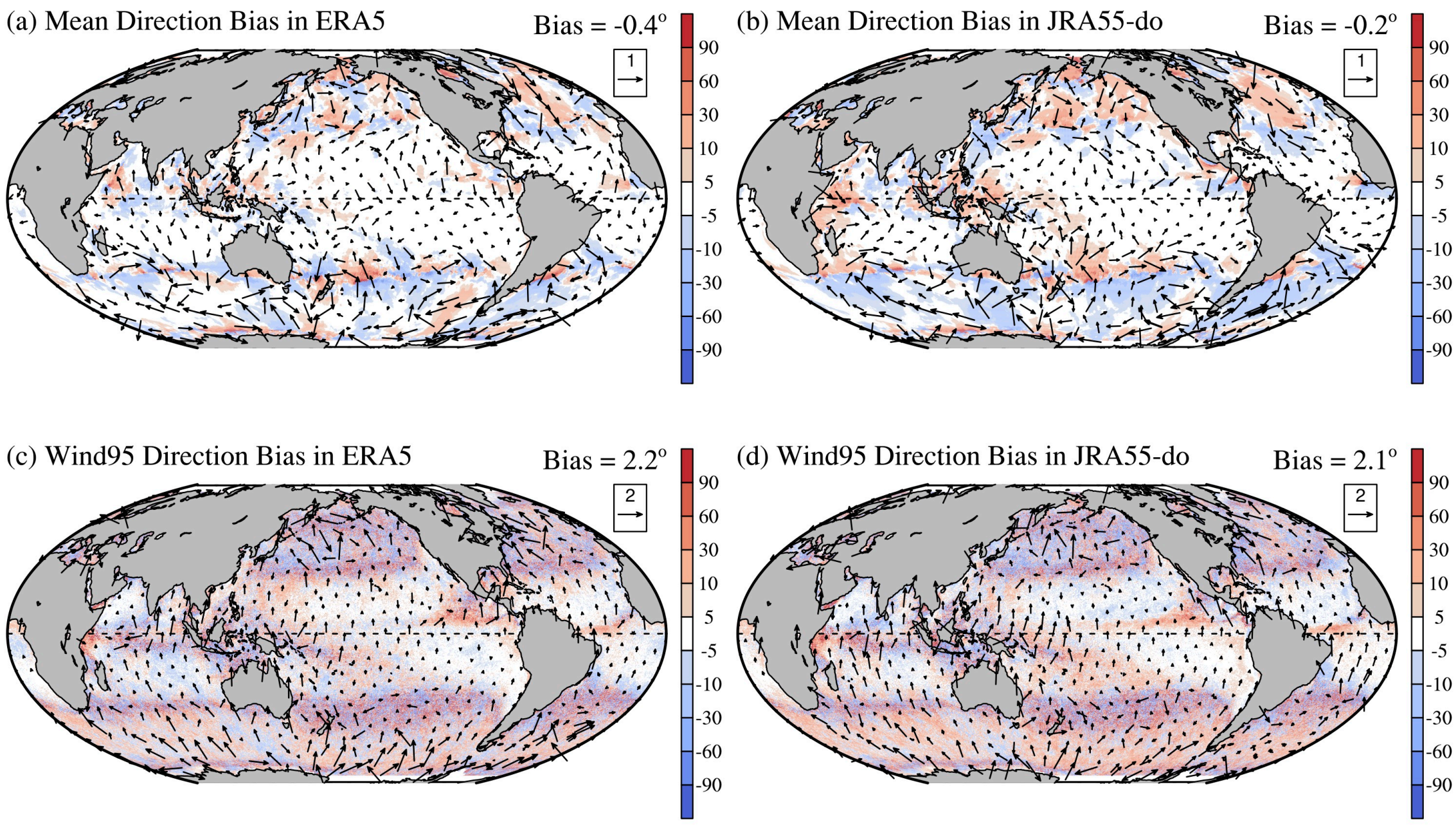

Fig. S11.

Wind-direction biases (°) of the (**a&b**) annual-mean and (**c&d**) 95th-percentile 10-m winds in ERA5 and JRA55-do relative to ASCAT during 2008-2020. Poor-quality ASCAT records affected by rain are removed. The reanalysis winds are interpolated to the along-track ASCAT locations. Shading represents wind direction bias, and positive (negative) values indicate clockwise (anticlockwise) deviations from ASCAT. Vector represents wind vector bias.

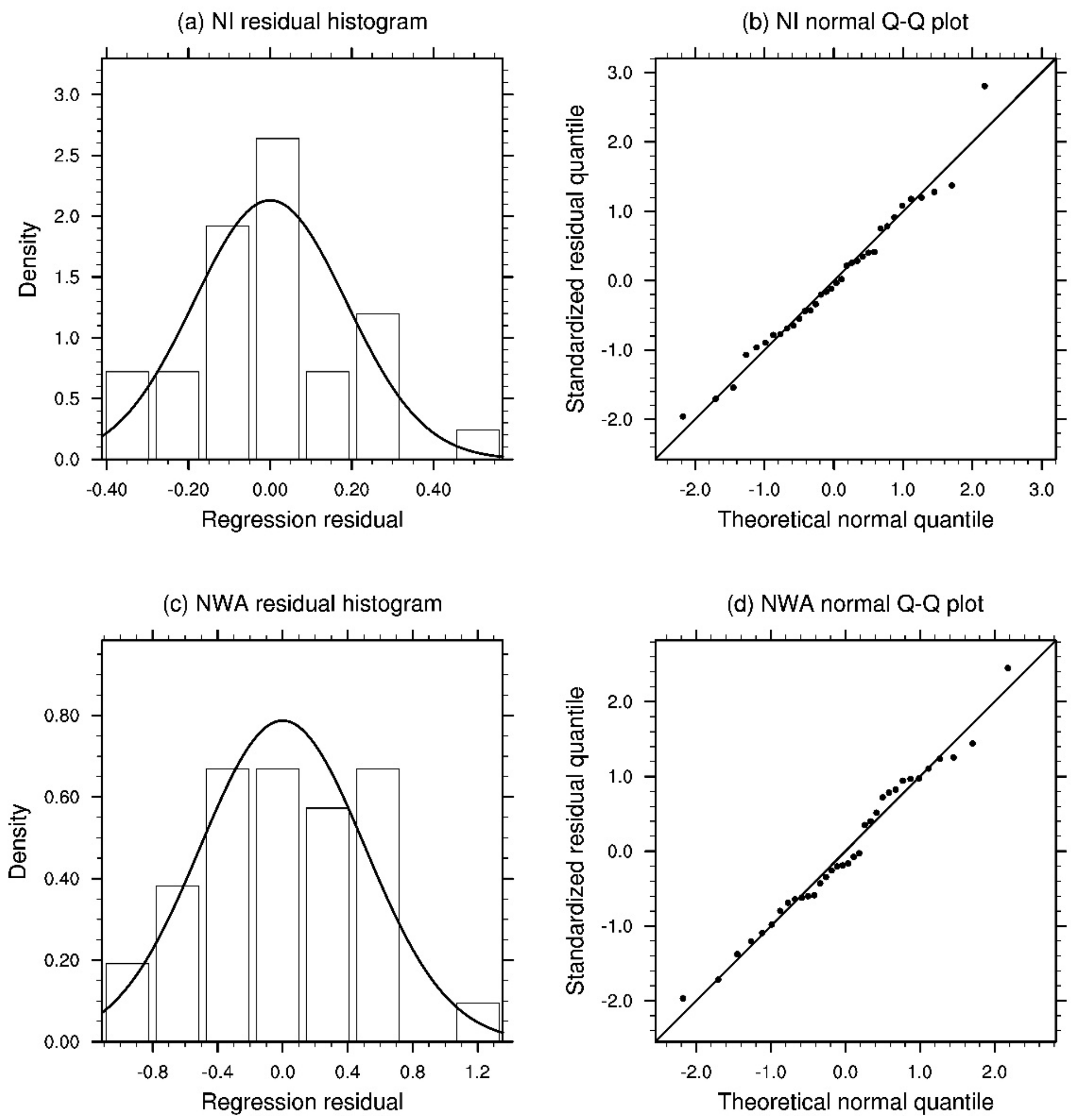

Fig. S12.

Residual diagnostics for the trends in annual differences between the "TCWIND" and "FILT" experiments over the North Indian Ocean (**a&b**) and the Northwest Atlantic (**c&d**). (**a&c**) Histograms show the regression residuals with fitted Gaussian distributions, and (**b&d**) quantile-quantile (Q-Q) plots compare standardized residuals with theoretical normal quantiles.

Table S1.

Area-averaged trends (per decade) in wave storm count, duration and significant wave height (SWH) for different basins. The upper part of the table reports the absolute magnitudes of the linear trends, while the lower part gives the corresponding percentage trends normalized by mean values. GL, NEP, NI, NWA, SWA, NEA, SEA, NWP, SP and SI represent the globe, the Northeast Pacific (180°W-90°W, 0-50°N), the North Indian Ocean (30°E-100°E, 0-30°N), the Northwest Atlantic (30°W-90°W, 0-50°N), the Southwest Atlantic (0-70°W, 0-40°S), the Northeast Atlantic (0-30°W, 0-50°N), the Southeast Atlantic (0-25°E, 0-40°S), the Northwest Pacific (100°E-180°E, 0-50°N), the South Pacific (135°E-70°W, 0-40°S) and the South Indian Ocean (25°E-135°E, 0-40°S), respectively. One and two asterisks indicate statistical significance at the 95% and 99% confidence levels, respectively, based on Student's t-test.

	**FILT Count (num/dec)**	**TCWIND Count (num/dec)**	**FILT Duration (hr/dec)**	**TCWIND Duration (hr/dec)**	**FILT SWH (m/dec)**	**TCWIND SWH (m/dec)**
**GL**	0.156 ± 0.066*	0.179 ± 0.070*	-0.197 ± 0.264	-0.355 ± 0.243	0.003 ± 0.002	0.003 ± 0.003
**NEP**	0.315 ± 0.257	0.433 ± 0.286	0.012 ± 0.383	-0.886 ± 0.373*	0.005 ± 0.006	0.011 ± 0.009
**NI**	-0.613 ± 0.130**	-0.549 ± 0.139**	-0.809 ± 1.451	-0.239 ± 1.354	0.001 ± 0.004	0.008 ± 0.006
**NWA**	0.311 ± 0.166	0.462 ± 0.180*	0.193 ± 0.350	0.269 ± 0.421	0.007 ± 0.007	0.012 ± 0.009
**SWA**	0.534 ± 0.193**	0.553 ± 0.194**	1.336 ± 0.500*	1.483 ± 0.628*	-0.001 ± 0.007	-0.001 ± 0.007
**NEA**	0.563 ± 0.313	0.569 ± 0.315	-0.438 ± 0.747	-0.401 ± 0.764	0.001 ± 0.015	-0.001 ± 0.015
**SEA**	0.352 ± 0.332	0.352 ± 0.331	0.254 ± 0.557	0.247 ± 0.558	0.010 ± 0.007	0.010 ± 0.007
**NWP**	0.094 ± 0.164	0.063 ± 0.167	-0.094 ± 0.567	-0.998 ± 0.490*	0.004 ± 0.006	0.008 ± 0.007
**SP**	0.232 ± 0.188	0.203 ± 0.197	-0.213 ± 0.890	-0.710 ± 0.863	-0.002 ± 0.003	-0.004 ± 0.006
**SI**	0.308 ± 0.224	0.295 ± 0.232	0.116 ± 0.862	-0.772 ± 0.815	-0.001 ± 0.004	-0.016 ± 0.006**
	**(%/dec)**	**(%/dec)**	**(%/dec)**	**(%/dec)**	**(%/dec)**	**(%/dec)**
**GL**	1.690 ± 0.710*	1.940 ± 0.760*	-0.470 ± 0.630	-0.840 ± 0.580	0.100 ± 0.080	0.100 ± 0.100
**NEP**	3.180 ± 2.610	4.380 ± 2.900	0.040 ± 1.190	-2.760 ± 1.160*	0.210 ± 0.230	0.430 ± 0.360
**NI**	-7.120 ± 1.510**	-6.380 ± 1.620**	-1.350 ± 2.420	-0.400 ± 2.260	0.040 ± 0.190	0.390 ± 0.300
**NWA**	3.300 ± 1.770	4.900 ± 1.910*	0.540 ± 0.980	0.750 ± 1.180	0.220 ± 0.230	0.380 ± 0.270
**SWA**	4.700 ± 1.700**	4.870 ± 1.700**	3.620 ± 1.360*	4.020 ± 1.700*	-0.020 ± 0.260	-0.020 ± 0.250
**NEA**	5.370 ± 2.990	5.430 ± 3.000	-1.100 ± 1.880	-1.010 ± 1.920	0.010 ± 0.390	-0.020 ± 0.390
**SEA**	2.950 ± 2.780	2.950 ± 2.780	0.750 ± 1.650	0.730 ± 1.650	0.270 ± 0.200	0.270 ± 0.200
**NWP**	1.170 ± 2.050	0.790 ± 2.090	-2.420 ± 1.380	-2.430 ± 1.190*	0.150 ± 0.240	0.320 ± 0.250
**SP**	2.670 ± 2.160	2.330 ± 2.270	-0.470 ± 1.970	-1.570 ± 1.910	-0.090 ± 0.110	-0.150 ± 0.220
**SI**	3.630 ± 2.640	3.490 ± 2.740	0.250 ± 1.830	-1.640 ± 1.730	-0.060 ± 0.160	-0.690 ± 0.250**
